# Temporal transcriptome and metabolite analyses provide insights into the biochemical and physiological processes underlying endodormancy release in pistachio (*Pistacia vera* L.) flower buds

**DOI:** 10.3389/fpls.2023.1240442

**Published:** 2023-09-22

**Authors:** Shu Yu, Douglas Amaral, Patrick H. Brown, Louise Ferguson, Li Tian

**Affiliations:** ^1^ Department of Plant Sciences, University of California, Davis, Davis, CA, United States; ^2^ University of California Cooperative Extension Kings County, Hanford, CA, United States

**Keywords:** pistachio, bud, dormancy, chilling accumulation, abscisic acid, NCED

## Abstract

Pistachio (*Pistacia vera* L.), an economically and nutritionally important tree crop, relies on winter chill for bud endodormancy break and subsequent blooming and nut production. However, insufficient winter chill poses an increasing challenge in pistachio growing regions. To gain a better understanding of the physiological and biochemical responses of endodormant pistachio buds to chilling accumulation, we investigated the global gene expression changes in flower buds of pistachio cv. Kerman that were cultivated at three different orchard locations and exposed to increasing durations of winter chill. The expression of genes encoding β-1,3-glucanase and β-amylase, enzymes responsible for breaking down callose (β-1,3-glucan) and starch (α-1,4-glucan), respectively, increased during the endodormancy break of pistachio buds. This result suggested that the breakdown of callose obstructing stomata as well as the release of glucose from starch enables symplasmic trafficking and provides energy for bud endodormancy break and growth. Interestingly, as chilling accumulation increased, there was a decrease in the expression of *nine-cis-epoxycarotenoid dioxygenase* (*NCED*), encoding an enzyme that uses carotenoids as substrates and catalyzes the rate-limiting step in abscisic acid (ABA) biosynthesis. The decrease in *NCED* expression suggests ABA biosynthesis is suppressed, thus reducing inhibition of endodormancy break. The higher levels of carotenoid precursors and a decrease in ABA content in buds undergoing endodormancy break supports this suggestion. Collectively, the temporal transcriptome and biochemical analyses revealed that the degradation of structural (callose) and non-structural (starch) carbohydrates, along with the attenuation of ABA biosynthesis, are critical processes driving endodormancy break in pistachio buds.

## 1 Introduction

Pistachio (*Pistacia vera* L.) is a dioecious, deciduous perennial tree grown in temperate regions. As with other temperate deciduous trees, such as apple, apricot, cherry, kiwi, and peach, pistachio buds enter a state of dormancy after the leaves drop in the fall. This process, known as winter dormancy, is essential for plant development and occurs in two sequential stages: endodormancy and ecodormancy (Lang et al., 1987). The progression of bud endodormancy is modulated by endogenous factors. Fulfilling the winter chill requirement (i.e. a specific duration of exposure to low temperatures) enables the release of buds from endodormancy, allowing the transition to ecodormancy. When environmental conditions become favorable, such as warmer temperatures and longer days, ecodormant buds exit their growth cessation state and visible bud break proceeds. As such, both the internal developmental program of the buds and external environmental factors play a role in the initiation and termination of winter dormancy phases, as well as the subsequent bud break in pistachio.

In recent years, an increasing occurrence of warm winters has led to insufficient winter chill that adversely affects flowering and pistachio yields by contributing to poor or non-uniform endodormant bud break. To overcome bud endodormancy caused by the lack of winter chill, bud-break enhancing chemicals, such as horticultural oil, kaolin clays, hydrogen cyanamide, and calcium carbonate, have been applied to pistachio trees. However, the effectiveness of these bud-break enhancing chemicals can be unpredictable due to various factors, such as the concentration of the chemicals, the timing of application, genetic variability among plant species and varieties, and the influence of environmental conditions (Guillamón et al., 2022). There is still much to be learned about the physiological and biochemical responses of endodormant pistachio buds to winter chill, and how bud-break enhancing chemicals function when the winter chill is inadequate.

Previous studies have linked the expression of *Dormancy Associated MADS-box* (*DAM*) transcription factors to the establishment and release of bud endodormancy in several plant species, including peach (*Prunus persica*) (Bielenberg et al., 2008; Jiménez et al., 2010; Yamane et al., 2011; Zhao et al., 2023), apple (*Malus* x *domestica*) (Porto et al., 2016), raspberry (*Rubus idaeus*) (Mazzitelli et al., 2007), Japanese apricot (*Prunus mume*) (Yamane et al., 2008), kiwifruit (*Actinidia deliciosa*) (Wu et al., 2012), Asian pear (*Pyrus pyrifolia*) (Ubi et al., 2010; Niu et al., 2016; Tuan et al., 2017; Li et al., 2021), European plum (*Prunus domestica*) (Quesada-Traver et al., 2020), sweet cherry (*Prunus avium*) (Wang et al., 2020), and blackcurrant (*Ribes nigrum*) (Hedley et al., 2010). When the winter chill requirement is met in the spring, *DAM* gene expression decreases, leading to the activation of genes that promote bud break and the resumption of growth. However, *DAM* gene expression remains high in warm winters where winter chill is insufficient, resulting in delayed or uneven bud break in the spring. Manipulating *DAM* gene expression has been shown to affect bud dormancy and growth in fruit trees. For example, overexpression of *MdDAMb* in transgenic apple (*Malus domestica*) trees prolonged dormancy and delayed bud break (Wu et al., 2017), while silencing *MdDAM1* led to the loss of dormancy in transgenic apple trees (Moser et al., 2020). The role of DAMs in regulating pistachio bud endodormancy has not been investigated.

In addition to DAM transcription factors, abscisic acid (ABA), a phytohormone derived from carotenoids, has also been implicated in the control of bud dormancy in perennial trees (Liu and Sherif, 2019; Pan et al., 2021). When treated with the inhibitor to carotenoid biosynthesis fluridone, carotenoid production was blocked and a higher percentage of bud break was observed in *in vitro* cultured rose (*Rosa hybrida*), suggesting a suppressive role of bud break by ABA (Bris et al., 1999). In a study conducted on grape (*Vitis vinifera*), application of a bud-break enhancing chemical, hydrogen cyanamide, lowered the expression of *nine-cis-epoxycarotenoid dioxygenase* (*NCED*) that encodes the cleavage enzyme for ABA biosynthesis and induced bud break (Zheng et al., 2015). In addition to ABA, other phytohormones, such as gibberellins, auxins, cytokinins, and jasmonic acid, have also been proposed to play a role in regulating bud dormancy in trees (Cooke et al., 2012).

To gain insights into the physiological and biochemical processes underlying bud endodormancy break in pistachio, we collected buds of pistachio cv. Kerman exposed to progressively increasing levels of winter chill through careful temperature monitoring and used them in transcriptome analysis to evaluate gene expression changes. To assess the impact of different environments on endodormancy break, buds for transcriptome analysis were collected from three orchard locations. Our analysis revealed that several genes exhibited endodormancy-associated expression, including *PvNCED3* for ABA biosynthesis, as well as two *β-1,3-glucanases* and a *β-amylase* for carbohydrate metabolism. The expression patterns revealed by transcriptome analysis were further validated by real-time quantitative PCR (qPCR) analysis. Additional carotenoid profiling and ABA analyses provided biochemical evidence for decreased ABA biosynthesis during bud endodormancy release.

## 2 Materials and methods

### 2.1 Plant materials

Female pistachio trees (*P. vera* cv. Kerman) located in three commercial orchards situated in Central California, including Couture in Kings County (36.050891, -120.012678), Rose in Kings County (35.904688, -119.911351), and Scroggs in Kern County (35.601124, -119.562749), were used to collect flower buds. The female pistachio cv. Kerman trees were interplanted with the male cv. Peters trees in all three orchards. HOBO^®^ temperature loggers (Onset Computer Corporation, Bourne, MA) were installed at the top of the canopy in each orchard to measure air temperature, humidity, and sunlight hours in the 2018-2019 growing season. The physiological status of flower buds, including their size (swelling and elongation), petal appearance, and the percentage that had bloomed, was monitored regularly.

The Dynamic Model was used to calculate chill portions (CPs), which indicates the accumulation of winter chill required for budbreak (Fishman et al., 1987). The yield-based chilling requirement for pistachio cv. Kerman was estimated to be 58 CPs or less based on previous modeling studies using historical data (Pope et al., 2015). For the transcriptome and metabolite analyses, three biological replicates of buds were collected at each time point for a wide range of CPs, including 45 CPs, 50 CPs, 55 CPs, 60 CPs, 65 CPs, and 70 CPs, which span flower bud dormancy development at each orchard location. Each biological replicate was composed of flower buds collected from five trees. These buds were detached from the branches using a razor blade and stored at -80°C. Before analysis, the frozen buds were ground into a fine powder in liquid nitrogen, and the resulting powder was divided into two aliquots for transcriptome and metabolite analyses.

### 2.2 Transcriptome analysis

Total RNA was extracted from ~50 mg of ground bud tissue using a cetyltrimethylammonium bromide (CTAB)-based method (Jaakola et al., 2001). Genomic DNA was removed from the extracted total RNA using the TURBO DNA-free™ kit (Invitrogen, Waltham, MA, USA). The cleaned total RNA was then divided into two aliquots for RNA-sequencing (RNA-seq) library construction and real-time qPCR analysis, respectively. For transcriptome analysis, mRNA in the total RNA sample was enriched using the oligo (dT) magnetic beads and the RNA-seq library was constructed using the Illumina TruSeq RNA Library Preparation Kit (Illumina, San Diego, CA, USA). The RNA-seq libraries were barcoded and subjected to paired-end 150 bp (PE150) sequencing on an Illumina NovaSeq6000 instrument (Illumina).

The raw sequence reads were processed by removing reads with adaptor sequences, low-quality reads, and reads with more than 10% N. The processed reads were then mapped to the fully sequenced pistachio genome (Zeng et al., 2019) using HISAT2, v.2.0.5 (Kim et al., 2019) and the read numbers mapped to each gene were counted using featureCounts (Liao et al., 2013). Fragments Per Kilobase of transcript per Million mapped reads (FPKM) values were then calculated for each gene based on the length of the gene and the reads count mapped to the gene. Analysis of differentially expressed genes was performed based on the mapped reads using DESeq2, v1.20.0, with *P*adj <= 0.05 (Anders and Huber, 2010).

Gene Ontology (GO) enrichment analysis of differentially expressed genes was conducted using the clusterProfiler R package (Wu et al., 2021), which corrects for gene length bias. GO terms with a corrected *p* value below 0.05 were considered significantly enriched by differentially expressed genes. The clusterProfiler R package was also used to test the statistical enrichment of differentially expressed genes in Kyoto Encyclopedia of Genes and Genomes (KEGG) pathways, which have frequently been used for understanding the molecular interactions and reaction networks in the biological system (Kanehisa et al., 2008).

### 2.3 Real-time qPCR analysis

Total RNA (1.2 μg) was used to synthesize first-strand cDNA (20 µl) using the iScript Advanced cDNA synthesis kit for RT-qPCR (BioRad, Hercules, CA, USA) following the manufacturer’s instructions. The expression levels of *PvNCED3* (*EVM0022332*), two *β-1,3-glucanase* genes (*EVM0025333* and *EVM0017922*), and a *β-amylase* gene (*EVM0000616*) in bud tissues were analyzed using the relative standard curve method (Applied Biosystems, 2008). The standard curve was constructed by bulking equal amounts of the cDNA synthesized from bud tissues collected from all locations and CPs. Real-time qPCR was performed in triplicate; each reaction contained 0.83 μl of the first-strand cDNA, 0.2 µM of each of the primers, and 10 μl of the iTaq™ Universal SYBR^®^ Green Supermix (BioRad). The expression of each gene was normalized using *β-tubulin* as a reference gene and calibrated by the average expression level of samples at 45 CPs for each location. The primers for *PvNCED3* and *β-tubulin* were previously reported (Moazzam Jazi et al., 2016; Moazzzam Jazi et al., 2017). The primer sequences for the *β-1,3-glucanase* genes, *EVM0025333* and *EVM0017922*, and the *β-amylase* gene, *EVM0000616*, are provided in **Table S1**. All primers were validated for amplification of a single product using the dissociation curve analysis.

### 2.4 Protein sequence and phylogenetic analysis

The Arabidopsis β-1,3-glucanase protein sequences were obtained from The Arabidopsis Information Resource (TAIR) using the Arabidopsis Genome Initiative (AGI) identifier numbers. The Arabidopsis and pistachio protein sequences were aligned using Multiple Sequence Comparison by Log-Expectation (MUSCLE) (Edgar, 2004) and the neighbor-joining phylogenetic tree was constructed using Molecular Evolutionary Genetics Analysis (MEGA) (Tamura et al., 2021) with 1,000 bootstrap tests. Glycosylphosphatidylinositol (GPI) anchoring sites in the β-1,3-glucanase protein sequences were predicted using PredGPI (Pierleoni et al., 2008). PfamScan was used for identifying functional domains in the protein sequences (Madeira et al., 2022).

### 2.5 Carotenoid and ABA analyses

Total carotenoids were extracted from ~50 mg of ground bud tissue using acetone:ethyl acetate (3:2, v/v) as previously described (Qin et al., 2016). Ten μl of the resulting carotenoid extract was injected onto a reverse-phase C_18_ column (Zorbax SB-C_18_, 150 mm x 4.6 mm, particle size 5 μm; Agilent, Santa Clara, CA, USA) and separated using an established gradient (Qin et al., 2012).

ABA was extracted from ~100 mg of ground pistachio bud tissue according to a previously published method with modification (Piskurewicz et al., 2009). The ABA was purified using a Sep-pak C_18_ reverse phase column (Waters, Milford, MA, USA) and quantified using the Phytodetek® Immunoassay Kit for ABA, following the manufacturer’s instructions with appropriate dilution.

Specifically, ABA was extracted from ground pistachio bud tissue using 1 ml of methanol: H_2_O: acetic acid (80:19:1, v/v/v) with 100 mg l^-1^ (w/v) butylated hydroxytoluene (BHT) overnight at 4°C with occasional vortexing. The extract was then re-extracted twice for 1 h with 0.5 ml of the same solvent. The combined extract was dried in a speed vac and dissolved in 1 ml of pre-chilled methanol: H_2_O: acetic acid (10:89:1, v/v/v) with 10 mg l^-1^ (w/v) BHT before loading onto a Sep-pak C_18_ reverse phase column. After washing with methanol: H_2_O: acetic acid (40:59:1, v/v/v) with 10 mg l^-1^ (w/v) BHT, ABA was eluted using methanol: H_2_O: acetic acid (80:19:1, v/v/v) with 100 mg l^-1^ (w/v) BHT and dried in a speed vac. The dried eluate was dissolved in 50 μl of methanol and diluted with 450 μl of Tris-buffered saline (TBS) before quantification using the Phytodetek® Immunoassay Kit for ABA. It should be noted that our preliminary analysis showed that unknown interfering substances affected the immunoassay for ABA quantification even after the cleanup with a Sep-pak C_18_ reverse phase column. To remove the interfering substances, 30 mg of insoluble polyvinylpyrrolidone (PVP) was added to each bud sample before the ABA extraction, as previously reported for the purification of ABA extracted from birch buds (Harrison and Saunders, 1975).

### 2.6 Promoter motif analysis

The genomic DNA sequences of *EVM0000616, EVM0025333*, *EVM0017922*, *EVM0022332*, *EVM0002551*, *EVM0022601*, *EVM0027471*, and *EVM0028749* were obtained from the Ensembl genomes database (Cunningham et al., 2021). Putative *cis*-elements within the 1,500 bp region upstream of the translational start (ATG) of the eight pistachio sequences were analyzed *in silico* using PlantCare (Lescot et al., 2002).

### 2.7 Statistical analysis

Statistical analysis was performed using Analysis of Variation (ANOVA) and the *post-hoc* Tukey’s Honest Significant Difference test. All statistical analyses were carried out using R (The R Foundation, Vienna, AT).

## 3 Results

### 3.1 Dormant flower buds with progressive chilling accumulation were collected by employing a dynamic model that utilizes temperatures in the growth environment

**Figure 1:**
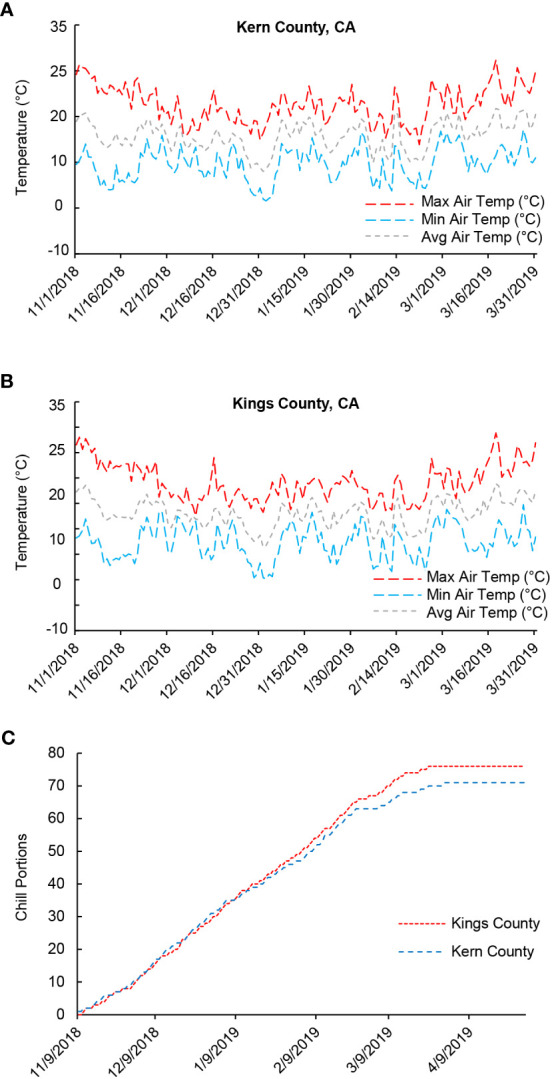
Air temperatures and chill portions (CPs) in Kern and Kings counties, California (CA) in the 2018-2019 growing season. **(A)** Average, minimum, and maximum daily air temperature (°C) in Kern County where the Scroggs orchard is located. **(B)** Average, minimum, and maximum daily temperature (°C) in Kings County where the Couture and Rose orchards are located. **(C)** Cumulative CPs in Kern and Kings counties.

To investigate the physiological and biochemical changes underlying bud endodormancy break, flower buds collected from the female pistachio cv. Kerman trees were analyzed. Trees from three different orchards located in two counties were used to determine if the growth environment, in addition to exposure to cold temperatures, may also impact bud endodormancy break. Temperature loggers installed in the orchards showed that the air temperature exhibited similar trends in both counties throughout the 2018-2019 growing season (**Figures 1A, B**). Moreover, the progression of CPs in Kern County and Kings County closely resembled each other, particularly during the phase of endodormancy release (**Figure 1C**). The relatively low average temperatures experienced during the 2018-2019 winter season facilitated the rapid accumulation of CPs in these pistachio buds. As a result, by late January/early February 2019, dormant buds in all three orchards had achieved the estimated threshold of 58 CPs required to ensure yield (**Figure 1C**) (Pope et al., 2015). For the transcriptome and metabolite analyses, flower buds were collected at the time points representing very early (45 CPs), early (50 CPs), near optimal (55 CPs), late (60 CPs), and very late (65 CPs and 70 CPs) stages of chilling accumulation.

### 3.2 Transcriptome analysis revealed genes involved in carbohydrate metabolism displaying expression patterns associated with the progression of bud endodormancy

To understand the molecular and physiological responses of pistachio flower buds to chilling accumulation, transcriptomes of flower buds collected at six different time points (45, 50, 55, 60, 65, and 70 CPs) were analyzed using RNA-seq. Approximately 30 million cleaned sequence reads (2 x 150 bp paired end) with a GC content around 44% and Q30 values about 94% were obtained for each transcriptome (**Table S2**). For all transcriptomes, 80%-89% of the cleaned sequence reads were mapped to the reference pistachio genome (Zeng et al., 2019) (**Table S3**). Interestingly, 86%-91% of expressed genes were shared across the three orchard locations at each CP (**Figure S1**), suggesting that they were likely controlled by the same genetic background of the pistachio trees cv. Kerman. On the other hand, genes that were uniquely expressed at each orchard location or shared by only two locations suggested that the growth environment could also impact gene expression (**Figure S1**).

**Figure 2:**
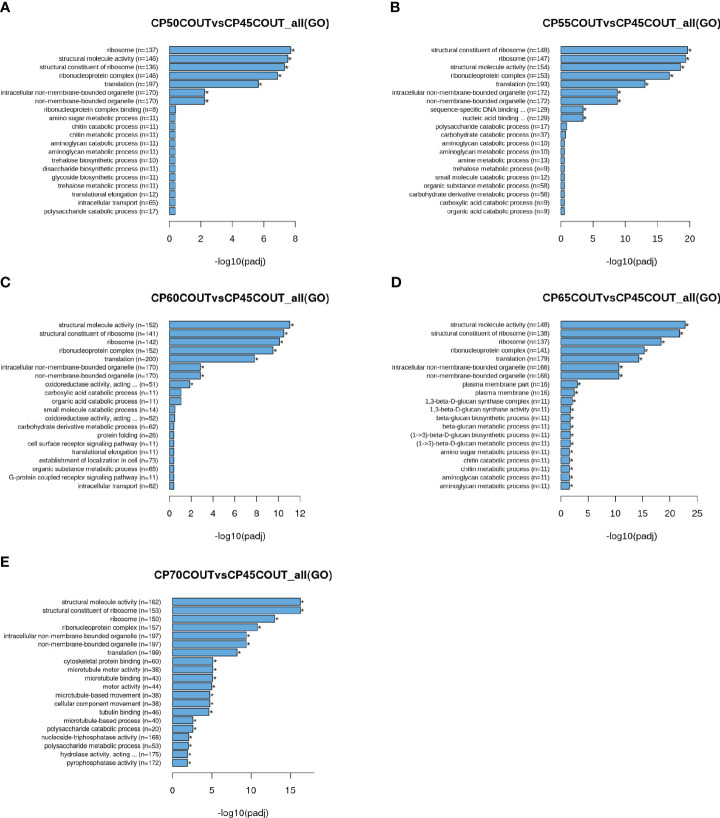
Gene ontology (GO) enrichment histograms for differentially expressed genes in buds collected at the Couture orchard. Top 20 significantly enriched terms in the GO enrichment analysis for each CP time point relative to 45 CPs are shown **(A–E)**.

**Figure 3:**
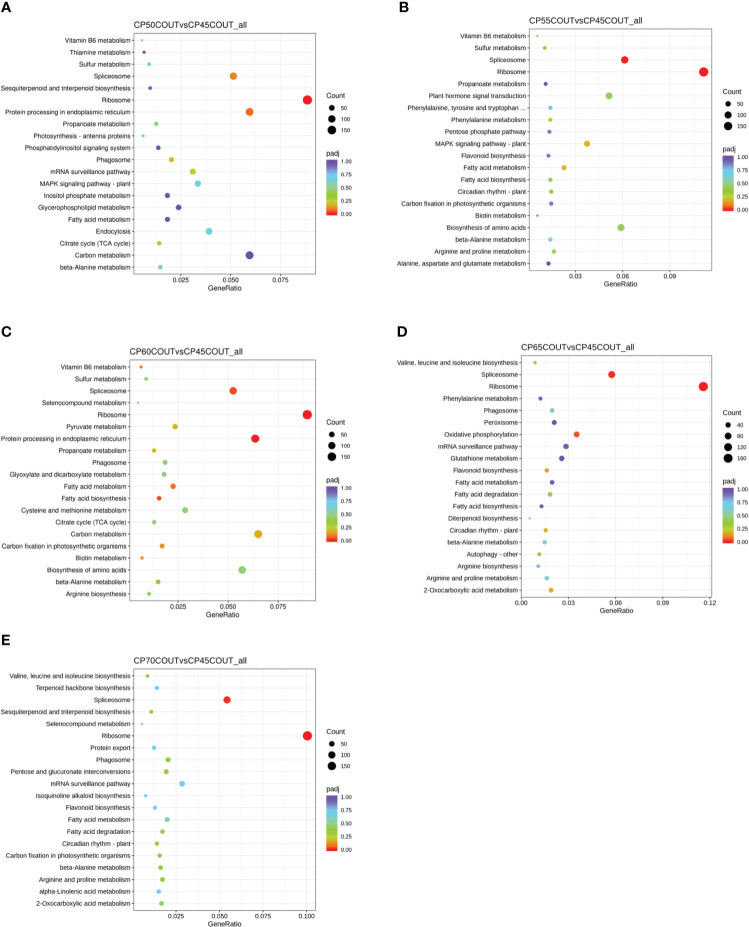
Kyoto Encyclopedia of Genes and Genomes (KEGG) pathway enrichment analysis for differentially expressed genes (DEGs) relative to 45 CPs in buds collected at the Couture orchard **(A–E)**. The size of the dot indicates the number of DEGs in the specific pathway, whereas the color of the dot represents the adjusted *p* value of the enrichment gene ratio (DEGs in the specific pathway/total DEGs).

To classify the function of differentially expressed genes induced by chilling accumulation, GO and KEGG pathway analyses were performed across all CPs (**Figures 2**, **3**, **S2-S5**). According to the GO terms, translation-related processes (including ribosomes, ribonucleoprotein complex, and translation) and carbohydrate metabolism are among the most differentially regulated biological processes during endodormancy progression and release (**Figures 2**, **S2**, and **S3**). Consistent with the GO analysis, significant enrichment in the KEGG pathways of ribosome, spliceosome, protein processing, and carbon metabolism was also observed in the pistachio flower buds transitioning from endodormancy to ecodormancy and bud break (**Figures 3**, **S4**, **S5**).

**Figure 4:**
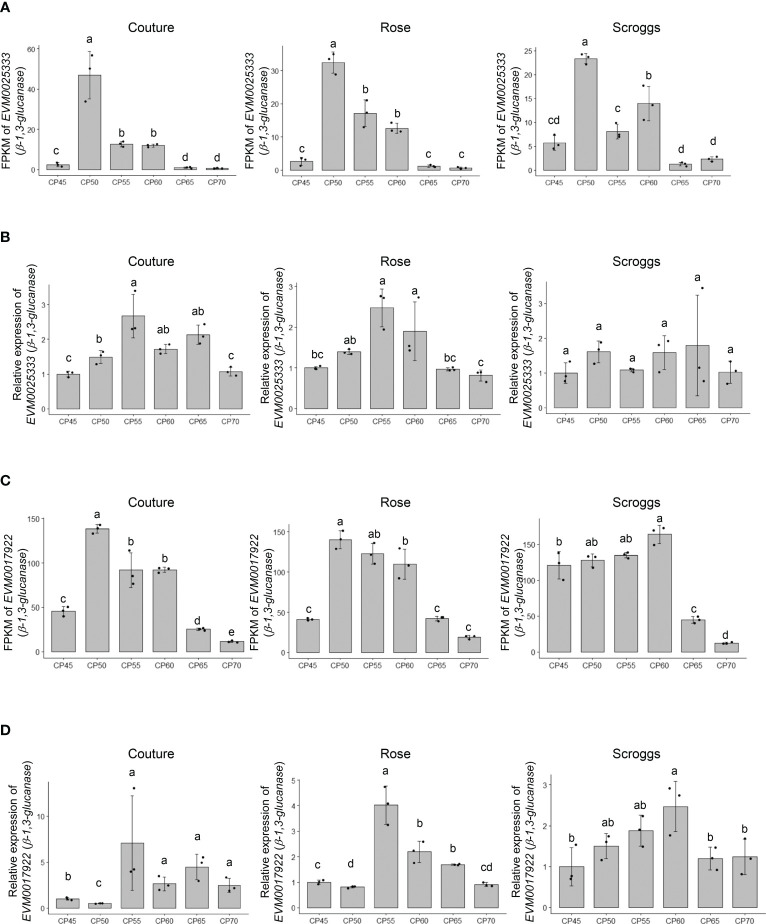
Expression analysis of the putative *β-1,3-glucanases EVM0025333* and *EVM0017922* in pistachio cv. Kerman buds exposed to different chill portions. The transcript levels were determined by RNA-Seq **(A)** and **(C)** as well as real-time qPCR **(B)** and **(D)**. Values are means with SD from three biological replicates. Different letters indicate statistically significant differences (*p* < 0.05) determined by Tukey’s Honest Significant Difference test. FPKM, Fragments Per Kilobase of transcript per Million mapped reads; CP, chill portion.

**Figure 5:**
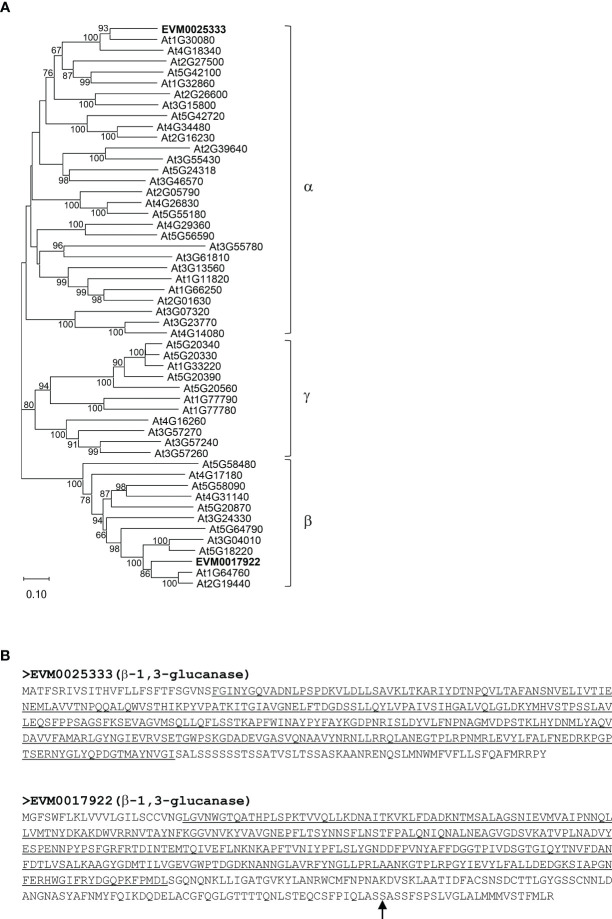
Phylogenetic and protein sequence analyses of the putative β-1,3-glucanases EVM0025333 and EVM0017922. **(A)** A neighbor-joining phylogenetic tree of 50 Arabidopsis β-1,3-glucanases as well as EVM0025333 and EVM0017922. **(B)** The protein sequences of EVM0025333 and EVM0017922. The conserved glycoside hydrolase family 17 domain is underlined. The glycosylphosphatidylinositol (GPI) anchor site in EVM0017922 is indicated with an arrow.

Among the genes involved in structural carbohydrate (e.g. cellulose and callose) metabolism (**Figure 2**), two genes encoding putative β-1,3-glucanases, EVM0025333 (396 amino acids) and EVM0017922 (478 amino acids), showed higher transcript levels at 50 CPs, 55 CPs, and 60 CPs compared to 45 CPs, 65 CPs, and 70 CPs in the RNA-seq analysis (**Figures 4A, C**). Similar patterns in transcript levels were confirmed through real-time qPCR analysis (**Figures 4B, D**). These expression patterns align with the breakdown of callose (β-1,3-glucan) by the activity of β-1,3-glucanases in buds undergoing progressive chilling accumulation and during the endodormancy release process. When the evolutionary relationships of EVM0025333 and EVM0017922 were explored with the distinct phylogenetic groups of Arabidopsis β-1,3-glucanases, α, β, and γ, EVM0025333 is closely associated with group α, while EVM0017922 clusters with group β of β-1,3-glucanases located in plasmodesmata (**Figure 5A**). The protein sequences of both EVM0025333 and EVM0017922 contain the glycoside hydrolase family 17 domain typically found in β-1,3-glucanases (**Figure 5B**). Furthermore, EVM0017922 also contains a GPI anchoring site that was predicted with high confidence (100%) (**Figure 5B**). The GPI anchoring site reportedly allows for post-translational modification of the protein for targeting to the plasma membrane (Zavaliev et al., 2016) and has previously been identified in β-1,3-glucanases located near plasmodesmata (Levy et al., 2007).

**Figure 6:**
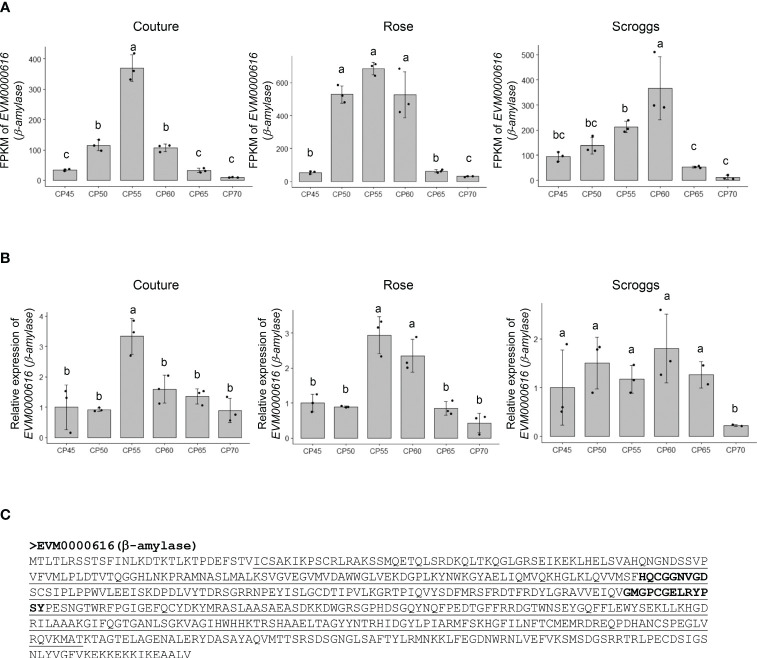
Relative expression of a putative *β-amylase EVM0000616* in pistachio cv. Kerman buds exposed to different chill portions. The expression levels were determined by RNA-Seq **(A)** and real-time qPCR **(B)** analyses. Values are means with SD from three biological replicates. Different letters indicate statistically significant differences (*p* < 0.05) determined by Tukey’s Honest Significant Difference test. FPKM, Fragments Per Kilobase of transcript per Million mapped reads; CP, chill portion **(C)** The protein sequence of EVM0000616 is shown with the conserved glycoside hydrolase family 14 domain underlined. The active sites in β-amylase sequences are highlighted in EVM0000616 in bold.

A notable change in genes involved in non-structural carbohydrate (e.g. soluble sugars and starch) metabolism is *EVM0000616* that encodes a putative β-amylase (549 amino acids), which catalyzes the breakdown of maltose into two glucose molecules at the α-1,4-glucosidic linkage (**Figure 6**). The RNA-seq and real-time qPCR analyses showed that the transcripts of *EVM0000616* accumulated most highly at 55 CPs and 60 CPs, but subsequently decreased at 65 CPs and 70 CPs (**Figures 6A, B**). While β-amylases can be found in either plastids or the cytosol, TargetP analysis did not identify a signal peptide in EVM0000616 for subcellular targeting (data not shown), suggesting that it is likely located in the cytosol. In addition to containing the glycoside hydrolase family 14 domain, EVM0000616 also possesses conserved active sites for β-amylases: including H-x-C-G-G-N-V-G-D and G-x-[SA]-G-E-[LIVM]-R-Y-P-S-Y (**Figure 6C**).

It is worth noting that the transcript levels of the three genes involved in non-structural and structural carbohydrate metabolism showed their highest expression at similar time points in buds collected from two different orchards: Rose and Couture orchards in Kings County (**Figures 4** and **6**). In contrast, buds collected from the Scroggs orchard, located in Kern County, exhibited a delayed peak in gene expression compared to those from the Rose and Couture orchards (**Figures 4** and **6**). These results suggested that, despite the geographical proximity of Kings and Kern counties within the Central Valley of California, the distinct microclimates in these regions had an environmental impact on the observed gene expression patterns.

### 3.3 A decrease in *PvNCED3* transcript levels was observed in buds undergoing endodormancy release, alongside concomitant increases in carotenoid precursors for ABA biosynthesis and decreases in ABA content

**Figure 7:**
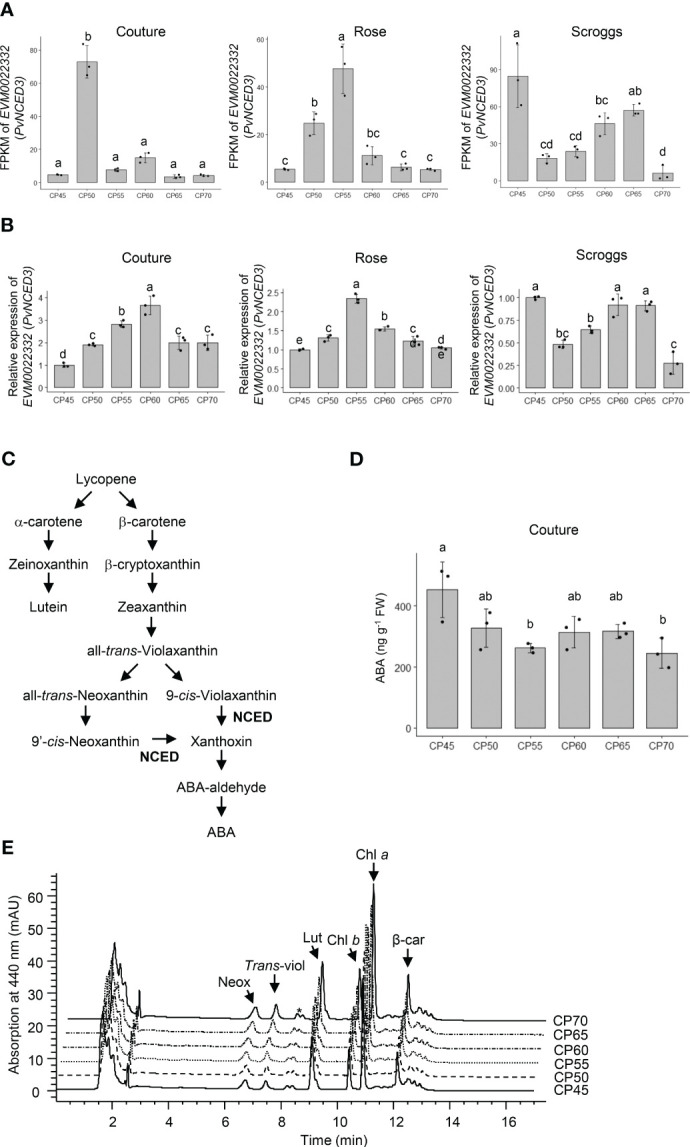
Relative expression of *PvNCED3* in pistachio cv. Kerman buds exposed to different chill portions. The expression levels were determined by RNA-Seq **(A)** and real-time qPCR **(B)** analyses. Values are means with SD from three biological replicates. Different letters indicate statistically significant differences (*p* < 0.05) determined by Tukey’s Honest Significant Difference test. FPKM, Fragments Per Kilobase of transcript per Million mapped reads; CP, chill portion. **(C)** A simplified pathway showing abscisic acid (ABA) biosynthesis from carotenoids in higher plants. NCED, nine-*cis*-epoxycarotenoid dioxygenase. **(D)** The ABA content (ng g^-1^ FW) of pistachio buds collected at the Couture orchard as determined by an immuno-based assay. FW, fresh weight. **(E)** Carotenoid profiles of pistachio buds collected at the Couture orchard. HPLC chromatograms of carotenoids extracted from pistachio buds exposed to different CPs. Neox, neoxanthin; *trans*-viol, *trans*-violaxanthin; Lut, lutein; Chl *b*, chlorophyll *b*; Chl *a*, chlorophyll *a*; β-car, β-carotene. * indicates an unidentified carotenoid.

In addition to the expression changes in *β-1,3-glucanases* and *β-amylase*, a clear trend of decreased expression of *PvNCED3* (*EVM0022332*) was observed in the transcriptome and real-time qPCR analyses of flower buds undergoing endodormancy release across all three orchard locations (**Figures 7A, B**). Because the NCED enzyme catalyzes a rate-limiting reaction in ABA biosynthesis (**Figure 7C**), the levels of ABA and its carotenoid biosynthetic precursors were also determined in the pistachio bud samples to assess the impact of *PvNCED3* expression changes (**Figures 7D, E**).

When the carotenoid profiles of pistachio flower buds were analyzed, neoxanthin, *trans*-violaxanthin, lutein, and β-carotene are the major carotenoids accumulating in this tissue (**Table 1**; **Figures 7E** and **S6**). Pistachio buds collected from the three orchards exhibited similar changes in carotenoid profiles through the progression of chilling accumulation. While the levels of lutein and β-carotene remained constant at all CPs analyzed, the amounts of *trans*-violaxanthin increased significantly (*p* < 0.05) at 65 CPs and 70 CPs relative to 45 CPs, 50 CPs, 55 CPs, and 60 CPs (**Table 1**; **Figures 7E** and **S6**). Elevated accumulation of neoxanthin was also observed in buds collected at 70 CPs relative to those collected at other CPs, particularly 60 CPs. Taken together, the increased accumulation of carotenoid precursors for ABA biosynthesis at 65 CPs and/or 70 CPs is consistent with a decreased expression of *PvNCED3* and less NCED activity for converting carotenoids to ABA (**Figures 7A, B**; **Table 1**). The ABA content in pistachio buds collected from the Couture orchard was also determined using an immuno-based assay, which is recognized as the most accessible method for measuring ABA concentrations in plant tissues (**Figure 7D**). The ABA content decreased rapidly during early to mid-chilling accumulation (45-55 CPs) and then remained relatively constant for 60-70 CPs (**Figure 7D**).

**Table 1:** Carotenoid content in pistachio cv. Kerman flower buds exposed to different chill portions.

Location	CPs	Neoxanthin	*trans*-Violaxanthin	Lutein	β-carotene
Couture	45	4.38 ± 0.35^b^	2.48 ± 0.24^b^	12.05 ± 0.44^a^	8.17 ± 0.27^a^
	50	4.49 ± 0.14^ab^	2.66 ± 0.14^b^	11.81 ± 0.06^a^	8.47 ± 1.49^a^
	55	4.28 ± 0.36^b^	2.79 ± 0.18^b^	11.86 ± 0.91^a^	8.84 ± 1.35^a^
	60	4.58 ± 0.52^ab^	2.96 ± 0.35^b^	12.00 ± 1.68^a^	8.87 ± 1.34^a^
	65	4.75 ± 0.14^ab^	3.82 ± 0.13^a^	12.27 ± 0.51^a^	7.99 ± 0.35^a^
	70	5.30 ± 0.21^a^	3.79 ± 0.24^a^	13.47 ± 0.74^a^	9.52 ± 0.96^a^
Rose	45	4.01 ± 0.24^ab^	2.24 ± 0.22^b^	12.65 ± 0.72^a^	9.24 ± 1.57^a^
	50	4.05 ± 0.34^ab^	2.33 ± 0.26^b^	11.68 ± 1.05^a^	9.15 ± 0.87^a^
	55	4.09 ± 0.39^ab^	2.63 ± 0.15^b^	12.65 ± 0.91^a^	10.66 ± 0.09^a^
	60	3.87 ± 0.11^b^	2.64 ± 0.24^b^	11.82 ± 0.55^a^	9.67 ± 0.73^a^
	65	4.52 ± 0.14^ab^	3.47 ± 0.26^a^	13.59 ± 0.48^a^	10.47 ± 0.39^a^
	70	4.66 ± 0.09^a^	3.62 ± 0.19^a^	13.57 ± 0.91^a^	10.95 ± 0.61^a^
Scroggs	45	3.57 ± 0.52^b^	2.04 ± 0.21^c^	11.55 ± 0.89^a^	8.22 ± 0.48^a^
	50	3.86 ± 0.27^b^	2.42 ± 0.19^bc^	10.78 ± 0.61^a^	8.18 ± 1.15^a^
	55	3.83 ± 0.08^b^	2.49 ± 0.07^bc^	11.05 ± 0.29^a^	8.97 ± 0.56^a^
	60	3.65 ± 0.49^b^	2.28 ± 0.35^c^	10.05 ± 1.52^a^	8.78 ± 0.95^a^
	65	4.27 ± 0.28^ab^	3.15 ± 0.19^b^	11.37 ± 0.90^a^	9.64 ± 0.90^a^
	70	5.47 ± 0.81^a^	5.27 ± 0.97^a^	10.85 ± 0.57^a^	8.84 ± 0.91^a^

Different letters indicate statistically significant differences (*p* < 0.05) for each carotenoid (nmol g^-1^ FW) in flower buds collected from the same location. The average and standard deviation of 3 biological replicates are presented. FW, fresh weight; CP, chill portion.

### 3.4 Expression patterns of *DAM* homologs were assessed through transcriptome analysis

To investigate the correlation between chilling accumulation and the expression of *DAM* homologs, four genes annotated as *DAM* homologs were identified from the transcriptome analysis: *EVM0002551*, *EVM0022601*, *EVM0027471*, and *EVM0028749* (**Figure S7**). Notably, *EVM0002551* and *EVM0022601*, despite being located on separate contigs, only differ in four nucleotides while sharing identical derived amino acid sequences (**Figures S7A, B**). In contrast to the candidate *β-1,3-glucanase* and *β-amylase* genes that exhibited peak expression levels around bud endodormancy release, *EVM0002551* and *EVM0022601* maintained similar levels of expression throughout 50-60 CPs, which were lower than both 45 CPs and 65 CPs (**Figures S7C, D**). The expression of *EVM0002551* and *EVM0022601* decreased at 70 CPs compared to 65 CPs at the three orchard locations, except for *EVM0022601* at the Rose orchard, which showed comparable expression for 65 CPs and 70 CPs (**Figures S7C, D**). The transcript levels of the other two *DAM* homologs, *EVM0027471* and *EVM0028749*, remained relatively constant from 45 CPs to 70 CPs, with the exception of *EVM0027471* at the Scroggs orchard, which showed a large decrease at 70 CPs (**Figures S7E, F**). These results collectively suggested that the four *DAM* homologs, *EVM0002551*, *EVM0022601*, *EVM0027471*, and *EVM0028749*, may operate through a different mode of action during the endodormancy release of pistachio buds as opposed to *DAM* genes in other plant species that typically exhibit a continued decrease in their transcript levels with exposure to low temperatures (Niu et al., 2016; Porto et al., 2016; Tuan et al., 2017; Quesada-Traver et al., 2020; Wang et al., 2020; Li et al., 2021; Zhao et al., 2023).

### 3.5 Potential regulation of the *β-1,3-glucanase*, *β-amylase*, *PvNCED3*, and *DAM* genes were explored by analyzing promoter sequences

**Figure 8:**
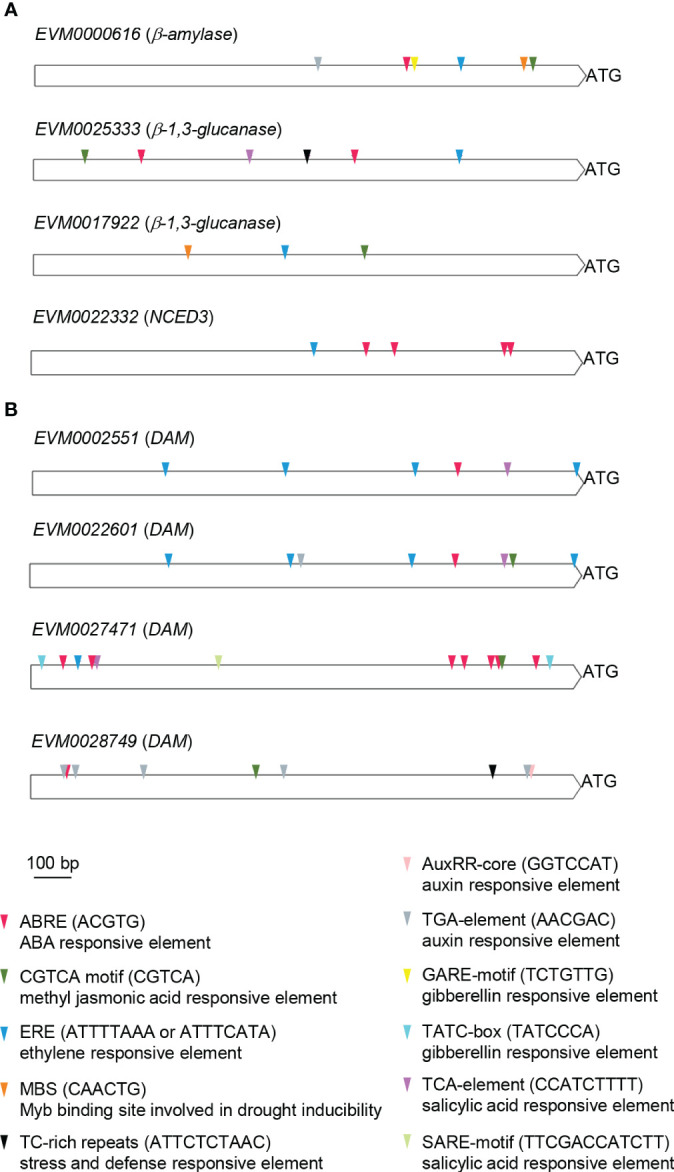
Promoter *cis*-element analysis of the putative *β-amylase*, *β-1,3-glucanase*, *PvNCED3*, and *DAM* genes. The 1,500 bp DNA sequences located upstream of the translational start (ATG) of **(A)** *β-amylase* (*EVM0000616*), *β-1,3-glucanases* (*EVM0025333*, *EVM0017922*), and *PvNCED3* (*EVM0022332*), as well as **(B)** *DAMs* (*EVM0002551*, *EVM0022601*, *EVM0027471*, *EVM0028749*) were analyzed using PlantCare. *Cis*-elements related to hormonal and stress responses are shown. ABRE, ABA responsive element; CGTCA, methyl jasmonic acid responsive element; ERE, ethylene responsive element; MBS, Myb binding site involved in drought inducibility; TC-rich repeats, stress and defense responsive element; AuxRR-core and TGA-element, auxin responsive elements; GARE and TATC-box, gibberellin responsive elements; SARE and TCA-element, salicylic acid responsive elements; bp, base pair.

To explore the regulatory control of the *β-amylase* (*EVM0000616*), *β-1,3-glucanase* (*EVM0025333, EVM0017922*), and *PvNCED3* (*EVM0022332*) genes associated with the progression of endodormancy release, as well as the *DAM* (*EVM0002551*, *EVM0022601*, *EVM0027471*, *EVM0028749*) genes which might play a role in bud endodormancy release, their promoter regions were searched for known *cis*-regulatory motifs (**Figure 8**). Notably, several hormonal and stress responsive elements were found within these promoters. For the *β-amylase*, *β-1,3-glucanase*, and *PvNCED3* genes, the ABRE (ABA responsive), ERE (ethylene responsive), and MeJA (CGTCA; methyl jasmonic acid responsive) motifs were present in all promoters, with the exception of the ABRE motif in *EVM0017922* (*β-1,3-glucanase*) and the MeJA motif in *PvNCED3* (*EVM0022332*) (**Figure 8A**). Additionally, the promoter regions of *EVM0000616* (*β-amylase*) and *EVM0017922* (*β-1,3-glucanase*) contained the MBS motif, which is associate with drought inducibility. Furthermore, the presence of the TCA motif, linked to salicylic acid response, and the TC-rich repeats known for its association with defense and stress response was observed in the promoter of *EVM0025333* (*β-1,3-glucanase*). On the other hand, the TGA element (auxin response) and GARE (gibberellin response) motifs were found in the promoter of *EVM0000616 (β-amylase*), suggesting regulatory mechanisms involving these two phytohormones (**Figure 8A**).

Among the four *DAM* genes (*EVM0002551*, *EVM0022601*, *EVM0027471*, *EVM0028749*), all featured ABRE, ERE, MeJA, and TCA motifs in their promoters, except for the lack of ERE and TCA motifs in *EVM0028749* and the absence of the MeJA motif in *EVM0002551* (**Figure 8B**). Interestingly, both *EVM0022601* and *EVM0028749* contained auxin responsive elements (AuxRR-core and/or TGA) in their promoters (**Figure 8B**). The TC-rich repeats associated with defense and stress response was only found in *EVM0028749*, while *EVM0027471* uniquely possessed the gibberellic acid responsive element (TATC-box) among the *DAM* genes analyzed (**Figure 8B**).

## 4 Discussion

By examining the correlation between gene expression changes and physiological processes during bud endodormancy break, our study revealed a coordinated response in structural and non-structural carbohydrates, as well as in the phytohormone ABA, during the exposure of pistachio flower buds to chilling temperatures. Specifically, we identified four genes (*PvNCED3*, one *β-amylase*, and two *β-1,3-glucanases*) that exhibited significant changes in transcript levels that correlated with the amount of chilling accumulation, suggesting their involvement in the progression and break of bud endodormancy in pistachio buds. Additionally, our analysis of carotenoid and ABA metabolites demonstrated alterations in both the precursors and products catalyzed by the NCED activity, thus aligning with the observed changes in *PvNCED3* gene expression. Furthermore, our comparison of gene expression changes in buds collected from three distinct orchard locations suggested an influence of the local growing environment on gene expression patterns.

In this study, the accurate measurement of chilling accumulation was achieved by installing weather stations within the orchard to closely monitor and record local weather conditions. In addition, the Dynamic Model, which considers both temperature fluctuations and the duration of cold temperatures, was used to calculate chilling accumulation (Fishman et al., 1987). Previous modeling studies have estimated chill requirements for pistachio cv. Kerman grown in the Central Valley of California (where the orchards in this study are located) using historical data spanning several decades. Analyses according to a bud-break-based (high percentage of bud-break on a shoot) suggested approximately 69 CPs for bud endodormancy break, whereas a nut yield-based (sustainable nut yield) method suggested approximately 58 CPs (Ferguson et al., 2008; Luedeling and Brown, 2011; Pope et al., 2015). However, it is worth noting that even when pistachio trees experience sufficient winter chill and bud-break, many flowers may fail to fully develop into split shell nuts due to resource competition or irrigation deficits. Therefore, it has been suggested that estimating the chill requirement based on the nut yield-based method is more appropriate than the bud-break-based method (Pope et al., 2015). As such, pistachio buds were collected in this study following the nut yield-based method, encompassing a range from low CPs (45 and 50 CPs) to near-optimal (55 CPs) and high CPs (60 CPs-70 CPs) for investigating endodormancy release.

DAM transcription factors have been reported for their roles in regulating the establishment and release of bud dormancy in tree crops (Liu and Sherif, 2019). There are often multiple copies of *DAM* genes in tree crops (Quesada-Traver et al., 2022). Four DAM homologs with the MADS motif were identified in our transcriptome analysis of pistachio flower buds; however, these pistachio homologs lack the 15-amino-acid DAM motif identified in other tree crops (**Figure S7**) (Quesada-Traver et al., 2022). Studies conducted on almond and peach have shown a gradual decrease in *DAM* expression during endodormancy release (Yamane et al., 2011; Prudencio Á et al., 2021). Interestingly, the expression of *DAM* homologs in pistachio buds did not follow a similar pattern (**Figure S7**). Among the four *DAM* homologs, *EVM0002551* and *EVM0022601* displayed more pronounced differential expression, though not showing a gradual decrease in expression, during chilling accumulation compared to *EVM0027471* and *EVM0028749*, which maintained relatively constant expression levels (**Figure S7**). The analysis of *cis*-elements in the promoters revealed a potential regulatory mechanism for pistachio *DAM* genes involving ABA, as the ABA responsive motif was identified in the promoters of all four pistachio *DAM* homologs (**Figure 8B**). Interestingly, despite the high similarity in expression patterns and identity in the encoded protein sequences between *EVM0002551* and *EVM0022601* (**Figure S7A**), the promoter region of *EVM0022601* contained auxin and MeJA responsive motifs, while *EVM0002551* did not (**Figure 8**). This suggests the possibility of distinct regulatory mechanisms directing their roles in various processes. Overall, the disparity in expression patterns for *DAM* genes in pistachios and other plant species emphasizes the need for further investigations to understand the specific mechanisms that govern the regulation of bud endodormancy in pistachios.

Previous research in several tree crops, such as grape, peach, and pear, established a strong correlation between endogenous ABA content and bud endodormancy, characterized by elevated ABA levels during early endodormancy, followed by a decline upon endodormancy release (Wang et al., 2002; Zheng et al., 2015; Li et al., 2018). The pattern of decreasing ABA content during endodormancy release was also evident in pistachio flower buds (**Figure 7D**), supporting the critical role of ABA in regulating the advancement of endodormancy in pistachios. This reduction in ABA levels can be at least partially attributed to a decrease in ABA biosynthesis, as shown by the decreased expression of the *PvNCED3* gene (**Figures 7A, B**). Moreover, during later stages of endodormancy, there was an increase in the levels of ABA precursors neoxanthin and violaxanthin (**Table 1**), further suggesting a decline in ABA production. However, in addition to biosynthesis, the processes of catabolism, transport, and signaling may also contribute to the regulation of ABA levels and its associated responses in pistachio buds, which could be a subject for future investigation.

In this study, we also explored the quantification of ABA concentrations in pistachio flower buds using an immuno-based assay (**Figure 7D**). It is worth noting that, in addition to following the standard protocol for ABA extraction from plant tissues, an additional step was incorporated when extracting ABA from pistachio bud samples. Insoluble PVP was introduced to the extraction buffer to effectively remove a large portion of unidentified substances that were observed to interfere with the subsequent immuno-based assay. However, it is possible that a minor fraction of the unidentified substances might have persisted in the extraction. Future investigations will explore the utilization of a liquid chromatography with tandem mass spectrometry (LC-MS/MS)-based method to separate and quantify ABA in pistachio buds, which was not possible in the present study given the limited availability of collected bud tissues (which also constrained the quantification of ABA for samples from the Rose and Scroggs orchards).

The role of ABA in controlling bud endodormancy involves the regulation of intercellular communication and metabolite exchange through the deposition and degradation of callose in plasmodesmata (Pan et al., 2021). In the early stage of endodormancy, ABA induces callose deposition at the plasmodesmata, leading to the obstruction of symplastic trafficking and reduced cellular activities. When exposed to chilling temperatures, callose gradually breaks down, allowing for the exchange of metabolites and signaling molecules through plasmodesmata, ultimately leading to bud endodormancy release (Rinne et al., 2001). In a previous phylogenetic analysis of 50 putative Arabidopsis β-1,3-glucanase proteins, three phylogenetic groups (α, β, and γ) were identified based on their evolutionary relationships (Doxey et al., 2007). Two pistachio *β-1,3-glucanase* genes, *EVM0025333* and *EVM0017922*, exhibited differential expression during bud endodormancy release, and their encoded proteins group with the Arabidopsis β-1,3-glucanase phylogenetic groups α and β, respectively (**Figure 5A**). Notably, EVM0017922 contains a predicted GPI anchor commonly found in plasmodesmata-associated β-1,3-glucanases (**Figure 5B**), suggesting its potential role in callose degradation during bud endodormancy release. Interestingly, *EVM0025333*, one of the *β-1,3-glucanase* genes with endodormancy-associated expression patterns, possesses an ABRE motif for ABA response in its promoter. In contrast, the ABRE motif is absent in the promoter of *EVM0017922* where *cis*-motifs that respond to phytohormones ethylene and MeJA were found (**Figure 8**). This result suggests that the regulation of endodormancy release-related callose breakdown at plasmodesmata likely involves a combination of ABA-dependent and ABA-independent mechanisms.

Another established process to induce bud dormancy in tree crops involves the accumulation of storage molecules, such as starch (α-1,4-glucan), in sink tissues (Tixier et al., 2019). When buds exit endodormancy, the breakdown of starch serves as an energy source for the resumption of growth. This breakdown process is enabled by a group of enzymes, including α-amylase, β-amylase, isoamylase, and starch debranching enzymes. In particular, β-amylase plays an important role in removing maltose from the non-reducing end of starch. The expression patterns of a *β-amylase* gene (*EVM0000616*) during the release of bud endodormancy in pistachio present compelling evidence supporting the involvement of starch degradation in this process (**Figure 6**). The promoter sequence of *EVM0000616* exhibits the presence of ABA, auxin, ethylene, gibberellin, and MeJA responsive motifs, suggesting its expression is likely regulated by a combination of multiple hormonal factors (**Figure 8**). Further investigations to explore the specific interactions and cross-talk between these hormonal pathways would provide insights into the regulatory mechanisms governing the expression of the putative *β-amylase* gene *EVM0000616*.

**Figure 9:**
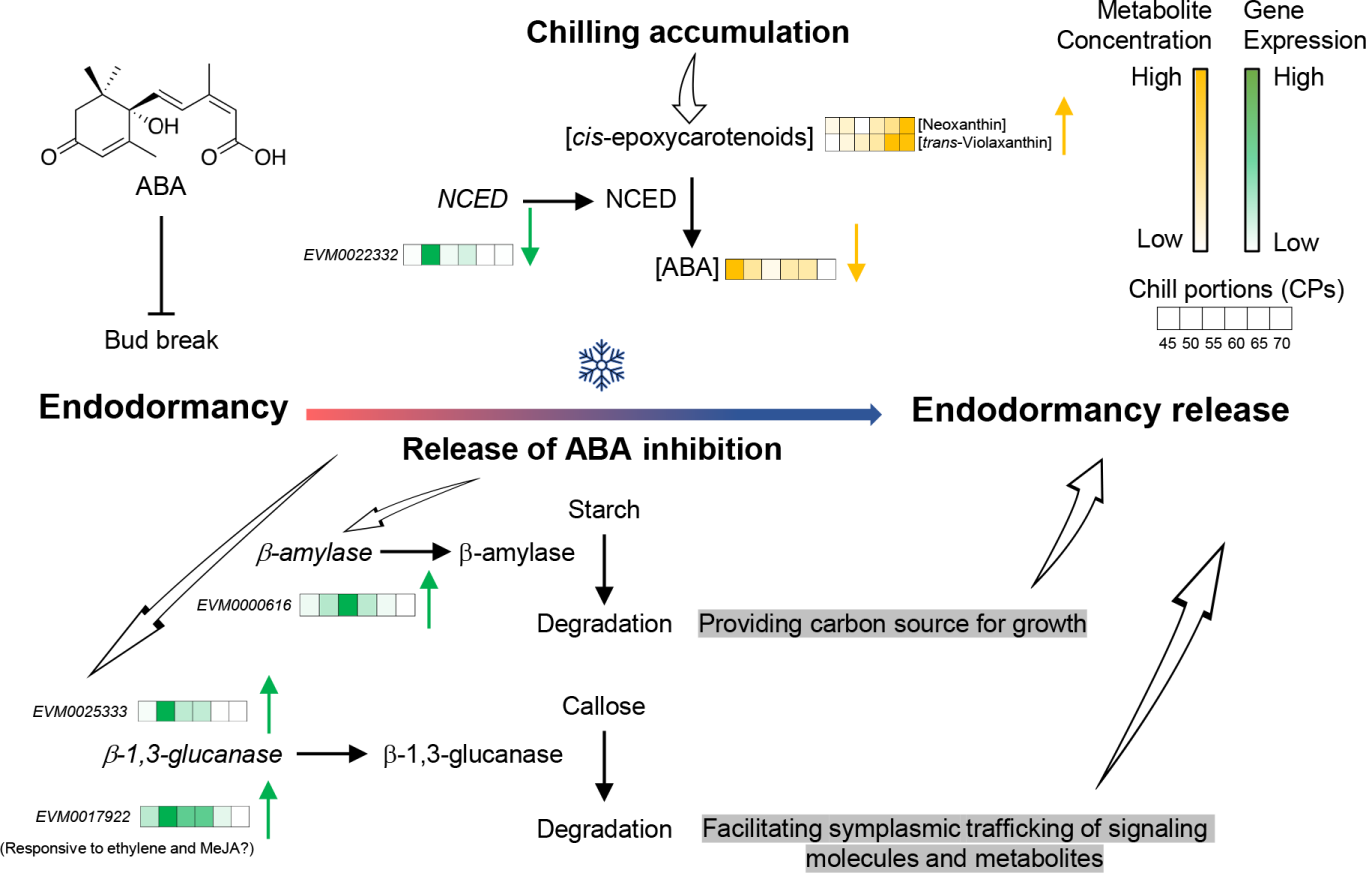
A proposed model for the physiological and biochemical processes underlying the release of endodormancy in pistachio buds. Fulfillment of chilling requirement represses the production of abscisic acid (ABA) in pistachio flower buds by suppressing the expression of *nine-cis-epoxycarotenoid dioxygenase* (*NCED*). This process results in a reduction in ABA levels and a concurrent rise in carotenoid precursors. A combination of ABA-dependent and ABA-independent mechanisms orchestrates the breakdown of callose at plasmodesmata, facilitating the intercellular exchange of molecules, and the degradation of starch to fuel cell growth.

Enhancing and synchronizing bud endodormancy release is crucial for successful flowering, pollination, and nut production in pistachio trees. The steady rise in global average surface temperatures in recent years amplifies the urgent need to address this challenge. Our study has provided valuable insights into the physiological and biochemical mechanisms involved in endodormancy release in pistachio buds, particularly the degradation of structural carbohydrates (callose) and non-structural carbohydrates (starch), as well as the modulation of ABA biosynthesis (**Figure 9**). This information can be used to devise strategies for enhancing pistachio production in regions with insufficient winter chill. For example, the development of gene expression markers that correlate with the fulfillment of chilling requirements could facilitate the creation of rapid and informative assays for selecting and applying budbreak enhancing chemicals. Our findings can also be utilized for comparative analysis with other perennial tree crops to unravel winter chill mechanisms to achieve controlled and uniform bud break, ensuring stable yields despite fluctuations in winter temperatures.

## Data availability statement

PRJNA984721 (the transcriptome data) has been released to public on the NCBI website.

## Author contributions

SY and DA contributed equally. SY, DA, PB, LF, and LT conceived and designed the experiments. DA did tissue collection. SY and DA performed the experiments. SY and LT analyzed the data. SY and LT wrote the manuscript. All authors read and approved the manuscript. All authors contributed to the article.
