# Supplementary material for: Temporal transcriptome and metabolite analyses provide insights into the biochemical and physiological processes underlying endodormancy release in pistachio (Pistacia vera L.) flower buds
Source: Front Plant Sci. 2023 Sep 22;14:1240442. doi: 10.3389/fpls.2023.1240442 (PMC10556704; doi:10.3389/fpls.2023.1240442)
Supplement: Supplementary file 1 [file Table_1.docx]

**Table S1** Sequences of primers used in the real-time qPCR analysis.

| Gene | Forward (5’🡪3’) | Reverse (5’🡪3’) |
| --- | --- | --- |
| *β-TUB* (*EVM0010714*) | TGGGACCCACGTGAAGTCAG | GAGTGGTGTAACTTGCTGCTTG |
| *PvNCED3* (*EVM0022332*) | CAACGCCGGACTGGTTTACT | TTTGCCCGAAACAGGATCAAC |
| *β-1,3-glucanase* (*EVM0025333*) | ACGAAATGTTGGCGGTTGTG | TTGTGGCGGGAACGTATGG |
| *β-1,3-glucanase* (*EVM0017922*) | TAAATGCCGATGTCTATG | ACTCAACAATCTGGGTCA |
| *β-amylase* (*EVM0000616*) | GCATTCCTCTACCACCAT | TCGTCCCGATCTGTCTGT |
