# Supplementary material for: Temporal transcriptome and metabolite analyses provide insights into the biochemical and physiological processes underlying endodormancy release in pistachio (Pistacia vera L.) flower buds
Source: Front Plant Sci. 2023 Sep 22;14:1240442. doi: 10.3389/fpls.2023.1240442 (PMC10556704; doi:10.3389/fpls.2023.1240442)
Supplement: Supplementary file 2 [file Table_2.docx]

**Table S2** Summary of RNA-seq data quality.

| Sample name | Raw reads | Clean reads | Raw bases | Clean bases | Error rate (%) | Q20 (%) | Q30 (%) | GC content (%) |
| --- | --- | --- | --- | --- | --- | --- | --- | --- |
| CP45COU1 | 33431539 | 32292384 | 10.0G | 9.7G | 0.03 | 97.86 | 94.01 | 45.34 |
| CP45COU2 | 34484731 | 33519813 | 10.3G | 10.1G | 0.03 | 97.69 | 93.71 | 44.35 |
| CP45COU3 | 30281662 | 29272863 | 9.1G | 8.8G | 0.03 | 97.76 | 93.95 | 44.56 |
| CP45ROS1 | 32130906 | 30944085 | 9.6G | 9.3G | 0.03 | 97.86 | 94.12 | 44.13 |
| CP45ROS2 | 31875453 | 30896191 | 9.6G | 9.3G | 0.03 | 97.68 | 93.78 | 44.44 |
| CP45ROS3 | 29700721 | 28195738 | 8.9G | 8.5G | 0.02 | 98.04 | 94.4 | 43.62 |
| CP45SCR1 | 32118471 | 30857114 | 9.6G | 9.3G | 0.02 | 98.02 | 94.39 | 43.97 |
| CP45SCR2 | 35879291 | 34428258 | 10.8G | 10.3G | 0.03 | 97.88 | 94.17 | 44.46 |
| CP45SCR3 | 34612188 | 33500287 | 10.4G | 10.1G | 0.03 | 97.86 | 94.02 | 44.23 |
| CP50COU1 | 38318219 | 36964565 | 11.5G | 11.1G | 0.03 | 97.9 | 94.13 | 44.58 |
| CP50COU2 | 35368304 | 34162195 | 10.6G | 10.2G | 0.03 | 97.89 | 94.14 | 44.09 |
| CP50COU3 | 38913755 | 37374529 | 11.7G | 11.2G | 0.03 | 97.15 | 92.43 | 44.15 |
| CP50ROS1 | 32614232 | 31542900 | 9.8G | 9.5G | 0.03 | 97.93 | 94.24 | 44.16 |
| CP50ROS2 | 33331682 | 32148267 | 10.0G | 9.6G | 0.03 | 97.89 | 94.12 | 44.02 |
| CP50ROS3 | 31263200 | 30062862 | 9.4G | 9.0G | 0.03 | 97.71 | 93.73 | 44.23 |
| CP50SCR1 | 60047168 | 57759530 | 18.0G | 17.3G | 0.02 | 98.34 | 95.23 | 44.22 |
| CP50SCR2 | 34434264 | 33174643 | 10.3G | 10.0G | 0.03 | 97.84 | 94.05 | 44.25 |
| CP50SCR3 | 29502220 | 28386881 | 8.9G | 8.5G | 0.03 | 97.76 | 93.91 | 44.15 |
| CP55COU1 | 29960944 | 28611729 | 9.0G | 8.6G | 0.03 | 97.94 | 94.14 | 43.71 |
| CP55COU2 | 34298989 | 32835347 | 10.3G | 9.9G | 0.03 | 97.92 | 94.17 | 44.01 |
| CP55COU3 | 38251451 | 36988361 | 11.5G | 11.1G | 0.03 | 97.86 | 94.11 | 44.56 |
| CP55ROS1 | 32386270 | 31152531 | 9.7G | 9.3G | 0.03 | 97.87 | 94.11 | 44.56 |
| CP55ROS2 | 34153087 | 32846249 | 10.2G | 9.9G | 0.03 | 97.75 | 93.85 | 44.42 |
| CP55ROS3 | 48429718 | 46524486 | 14.5G | 14.0G | 0.03 | 97.82 | 94 | 44.37 |
| CP55SCR1 | 32763678 | 31580569 | 9.8G | 9.5G | 0.03 | 97.73 | 93.77 | 44.35 |
| CP55SCR2 | 29911688 | 28680229 | 9.0G | 8.6G | 0.03 | 97.79 | 93.93 | 44.29 |
| CP55SCR3 | 34690814 | 33566886 | 10.4G | 10.1G | 0.02 | 97.99 | 94.49 | 44.29 |
| CP60COU1 | 33517333 | 32375377 | 10.1G | 9.7G | 0.02 | 98 | 94.38 | 44.45 |
| CP60COU2 | 31332803 | 30262591 | 9.4G | 9.1G | 0.03 | 97.93 | 94.21 | 44.2 |
| CP60COU3 | 34196746 | 33013371 | 10.3G | 9.9G | 0.03 | 97.92 | 94.2 | 44.28 |
| CP60ROS1 | 29967112 | 28586572 | 9.0G | 8.6G | 0.02 | 98.03 | 94.36 | 43.65 |
| CP60ROS2 | 36358710 | 35011247 | 10.9G | 10.5G | 0.03 | 97.84 | 94.04 | 43.79 |
| CP60ROS3 | 36938307 | 35475234 | 11.1G | 10.6G | 0.03 | 97.8 | 94 | 44.3 |
| CP60SCR1 | 34242176 | 33133118 | 10.3G | 9.9G | 0.03 | 97.88 | 94.07 | 44.56 |
| CP60SCR2 | 33972286 | 32751062 | 10.2G | 9.8G | 0.03 | 97.79 | 93.91 | 44.51 |
| CP60SCR3 | 36371362 | 34979872 | 10.9G | 10.5G | 0.03 | 97.93 | 94.24 | 44.37 |
| CP65COU1 | 31536567 | 30397161 | 9.5G | 9.1G | 0.03 | 97.32 | 92.52 | 44.15 |
| CP65COU2 | 32296183 | 31146732 | 9.7G | 9.3G | 0.02 | 98 | 94.35 | 44.14 |
| CP65COU3 | 32724889 | 31602431 | 9.8G | 9.5G | 0.02 | 97.95 | 94.25 | 44.26 |
| CP65ROS1 | 32447745 | 31278181 | 9.7G | 9.4G | 0.03 | 97.9 | 94.18 | 44.44 |
| CP65ROS2 | 33391404 | 32267822 | 10.0G | 9.7G | 0.03 | 97.76 | 93.79 | 44.11 |
| CP65ROS3 | 32538366 | 31129965 | 9.8G | 9.3G | 0.02 | 97.93 | 94.35 | 44.37 |
| CP65SCR1 | 33122916 | 31296578 | 9.9G | 9.4G | 0.02 | 98.14 | 94.56 | 43.36 |
| CP65SCR2 | 36029701 | 35032396 | 10.8G | 10.5G | 0.03 | 97.94 | 94.22 | 44.16 |
| CP65SCR3 | 34611102 | 33562811 | 10.4G | 10.1G | 0.03 | 97.94 | 94.17 | 44.12 |
| CP70COU1 | 33818535 | 32765352 | 10.1G | 9.8G | 0.03 | 97.9 | 93.99 | 43.94 |
| CP70COU2 | 36926403 | 35952261 | 11.1G | 10.8G | 0.02 | 98.1 | 94.57 | 44.2 |
| CP70COU3 | 30437393 | 29480370 | 9.1G | 8.8G | 0.03 | 97.95 | 94.16 | 44.31 |
| CP70ROS1 | 32968696 | 31696682 | 9.9G | 9.5G | 0.02 | 98.1 | 94.57 | 44.1 |
| CP70ROS2 | 31582778 | 30547951 | 9.5G | 9.2G | 0.02 | 98.01 | 94.36 | 44.2 |
| CP70ROS3 | 30957611 | 29911837 | 9.3G | 9.0G | 0.03 | 97.85 | 94.02 | 44.2 |
| CP70SCR1 | 32518629 | 31869536 | 9.8G | 9.6G | 0.02 | 98.22 | 94.7 | 44.09 |
| CP70SCR2 | 33711283 | 32552979 | 10.1G | 9.8G | 0.02 | 98.26 | 94.74 | 43.63 |
| CP70SCR3 | 36199207 | 35202080 | 10.9G | 10.6G | 0.02 | 98.17 | 94.57 | 43.92 |
