# Supplementary material for: Temporal transcriptome and metabolite analyses provide insights into the biochemical and physiological processes underlying endodormancy release in pistachio (Pistacia vera L.) flower buds
Source: Front Plant Sci. 2023 Sep 22;14:1240442. doi: 10.3389/fpls.2023.1240442 (PMC10556704; doi:10.3389/fpls.2023.1240442)
Supplement: Supplementary file 3 [file Table_3.docx]

**Table S3** Summary of RNA-seq reads mapping results.

| Sample name | Total reads | Total mapped reads | Uniquely mapped reads | Multiple mapped reads | Total mapping rate | Uniquely mapping rate | Multiple mapping rate |
| --- | --- | --- | --- | --- | --- | --- | --- |
| CP45COU1 | 64584768 | 51633370 | 44177604 | 7455766 | 79.95% | 68.40% | 11.54% |
| CP45COU2 | 67039626 | 55356813 | 47323107 | 8033706 | 82.57% | 70.59% | 11.98% |
| CP45COU3 | 58545726 | 47219296 | 40296728 | 6922568 | 80.65% | 68.83% | 11.82% |
| CP45ROS1 | 61888170 | 51482772 | 44007703 | 7475069 | 83.19% | 71.11% | 12.08% |
| CP45ROS2 | 61792382 | 50262558 | 42925746 | 7336812 | 81.34% | 69.47% | 11.87% |
| CP45ROS3 | 56391476 | 46553369 | 39863557 | 6689812 | 82.55% | 70.69% | 11.86% |
| CP45SCR1 | 61714228 | 52706502 | 45056513 | 7649989 | 85.40% | 73.01% | 12.40% |
| CP45SCR2 | 68856516 | 57554924 | 49039819 | 8515105 | 83.59% | 71.22% | 12.37% |
| CP45SCR3 | 67000574 | 57101605 | 48749975 | 8351630 | 85.23% | 72.76% | 12.47% |
| CP50COU1 | 73929130 | 63723379 | 53997610 | 9725769 | 86.20% | 73.04% | 13.16% |
| CP50COU2 | 68324390 | 59453028 | 50572317 | 8880711 | 87.02% | 74.02% | 13.00% |
| CP50COU3 | 74749058 | 64787742 | 55119758 | 9667984 | 86.67% | 73.74% | 12.93% |
| CP50ROS1 | 63085800 | 54681311 | 46420801 | 8260510 | 86.68% | 73.58% | 13.09% |
| CP50ROS2 | 64296534 | 55904643 | 47628471 | 8276172 | 86.95% | 74.08% | 12.87% |
| CP50ROS3 | 60125724 | 51987311 | 44192869 | 7794442 | 86.46% | 73.50% | 12.96% |
| CP50SCR1 | 1.16E+08 | 1E+08 | 85064950 | 15106039 | 86.71% | 73.64% | 13.08% |
| CP50SCR2 | 66349286 | 57468431 | 48930324 | 8538107 | 86.61% | 73.75% | 12.87% |
| CP50SCR3 | 56773762 | 49010882 | 41724920 | 7285962 | 86.33% | 73.49% | 12.83% |
| CP55COU1 | 57223458 | 49686043 | 42093688 | 7592355 | 86.83% | 73.56% | 13.27% |
| CP55COU2 | 65670694 | 57158756 | 48431350 | 8727406 | 87.04% | 73.75% | 13.29% |
| CP55COU3 | 73976722 | 63911792 | 54147155 | 9764637 | 86.39% | 73.19% | 13.20% |
| CP55ROS1 | 62305062 | 53917683 | 45726227 | 8191456 | 86.54% | 73.39% | 13.15% |
| CP55ROS2 | 65692498 | 56608180 | 47867404 | 8740776 | 86.17% | 72.87% | 13.31% |
| CP55ROS3 | 93048972 | 80806892 | 68476474 | 12330418 | 86.84% | 73.59% | 13.25% |
| CP55SCR1 | 63161138 | 54373894 | 46236415 | 8137479 | 86.09% | 73.20% | 12.88% |
| CP55SCR2 | 57360458 | 49817755 | 42308241 | 7509514 | 86.85% | 73.76% | 13.09% |
| CP55SCR3 | 67133772 | 58102348 | 49355203 | 8747145 | 86.55% | 73.52% | 13.03% |
| CP60COU1 | 64750754 | 56345181 | 47824678 | 8520503 | 87.02% | 73.86% | 13.16% |
| CP60COU2 | 60525182 | 52310707 | 44537549 | 7773158 | 86.43% | 73.59% | 12.84% |
| CP60COU3 | 66026742 | 57382924 | 48752642 | 8630282 | 86.91% | 73.84% | 13.07% |
| CP60ROS1 | 57173144 | 49727240 | 42236650 | 7490590 | 86.98% | 73.87% | 13.10% |
| CP60ROS2 | 70022494 | 60487622 | 51609177 | 8878445 | 86.38% | 73.70% | 12.68% |
| CP60ROS3 | 70950468 | 60889636 | 51779505 | 9110131 | 85.82% | 72.98% | 12.84% |
| CP60SCR1 | 66266236 | 57008795 | 48446185 | 8562610 | 86.03% | 73.11% | 12.92% |
| CP60SCR2 | 65502124 | 56571318 | 47953915 | 8617403 | 86.37% | 73.21% | 13.16% |
| CP60SCR3 | 69959744 | 60385135 | 51317119 | 9068016 | 86.31% | 73.35% | 12.96% |
| CP65COU1 | 60794322 | 51867845 | 44181209 | 7686636 | 85.32% | 72.67% | 12.64% |
| CP65COU2 | 62293464 | 53234655 | 45264087 | 7970568 | 85.46% | 72.66% | 12.80% |
| CP65COU3 | 63204862 | 54080169 | 45994930 | 8085239 | 85.56% | 72.77% | 12.79% |
| CP65ROS1 | 62556362 | 52612367 | 44768962 | 7843405 | 84.10% | 71.57% | 12.54% |
| CP65ROS2 | 64535644 | 55003297 | 46967805 | 8035492 | 85.23% | 72.78% | 12.45% |
| CP65ROS3 | 62259930 | 52767802 | 44912206 | 7855596 | 84.75% | 72.14% | 12.62% |
| CP65SCR1 | 62593156 | 54136398 | 46176760 | 7959638 | 86.49% | 73.77% | 12.72% |
| CP65SCR2 | 70064792 | 60301773 | 51315793 | 8985980 | 86.07% | 73.24% | 12.83% |
| CP65SCR3 | 67125622 | 57888721 | 49372051 | 8516670 | 86.24% | 73.55% | 12.69% |
| CP70COU1 | 65530704 | 56235365 | 47786589 | 8448776 | 85.82% | 72.92% | 12.89% |
| CP70COU2 | 71904522 | 61395633 | 52111386 | 9284247 | 85.38% | 72.47% | 12.91% |
| CP70COU3 | 58960740 | 49943834 | 42443283 | 7500551 | 84.71% | 71.99% | 12.72% |
| CP70ROS1 | 63393364 | 54184418 | 45968859 | 8215559 | 85.47% | 72.51% | 12.96% |
| CP70ROS2 | 61095902 | 52556337 | 44701354 | 7854983 | 86.02% | 73.17% | 12.86% |
| CP70ROS3 | 59823674 | 50893026 | 43342565 | 7550461 | 85.07% | 72.45% | 12.62% |
| CP70SCR1 | 63739072 | 56501430 | 47682827 | 8818603 | 88.64% | 74.81% | 13.84% |
| CP70SCR2 | 65105958 | 58241727 | 49103375 | 9138352 | 89.46% | 75.42% | 14.04% |
| CP70SCR3 | 70404160 | 61975596 | 52347827 | 9627769 | 88.03% | 74.35% | 13.68% |
