# Supplementary material for: Temporal transcriptome and metabolite analyses provide insights into the biochemical and physiological processes underlying endodormancy release in pistachio (Pistacia vera L.) flower buds
Source: Front Plant Sci. 2023 Sep 22;14:1240442. doi: 10.3389/fpls.2023.1240442 (PMC10556704; doi:10.3389/fpls.2023.1240442)
Supplement: Supplementary file 4 [file Presentation_1.pdf]

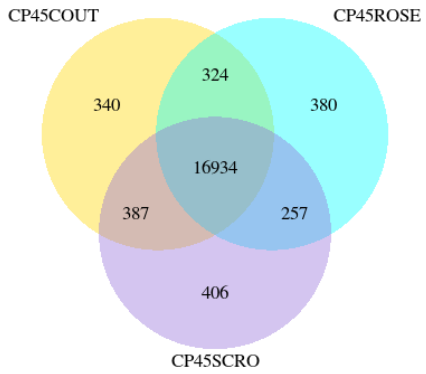

16934 = 88.99% of 19028 genes collectively expressed at CP45 at the three orchard locations

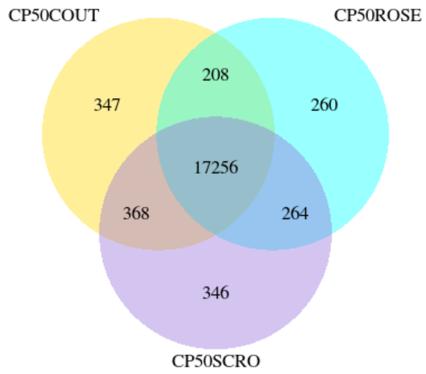

17256 = 90.59% of 19049 genes collectively expressed at CP50 at the three orchard locations

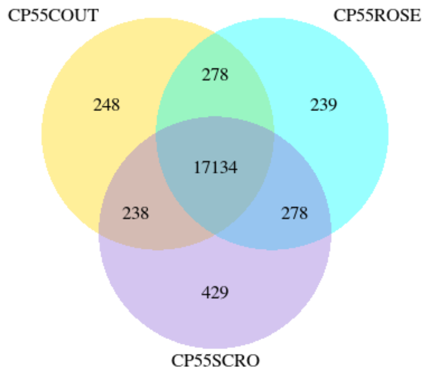

17134 = 90.93% of 18844 genes collectively expressed at CP55 at the three orchard locations

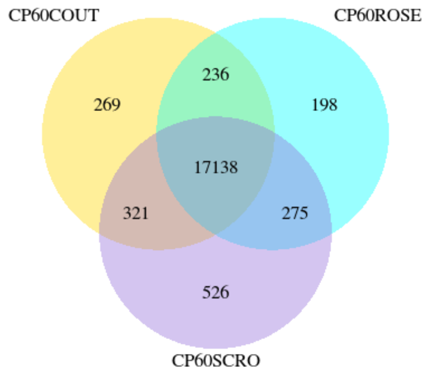

17138 = 90.38% of 18963 genes collectively expressed at CP60 at the three orchard locations

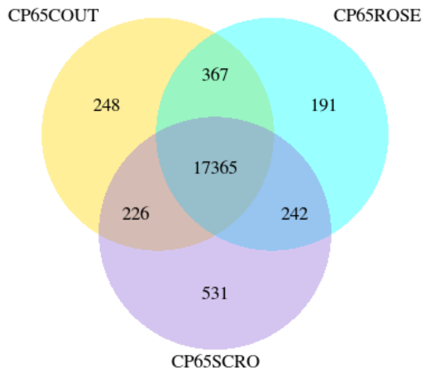

17365 = 90.58% of 19170 genes collectively expressed at CP65 at the three orchard locations

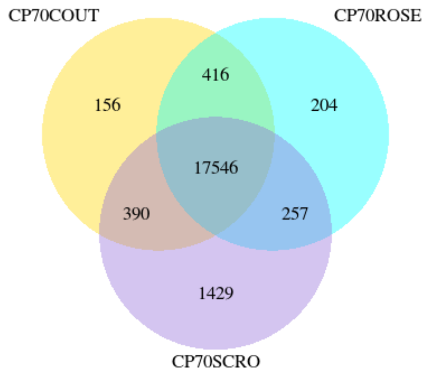

17546 = 86.02% of 20398 genes collectively expressed at CP70 at the three orchard locations

Figure S1. Coexpression Venn diagram depicting the number of genes that are uniquely expressed within each orchard location, with the overlapping regions showing the number of genes that are co-expressed in two or more orchard locations.
