# Supplementary material for: Temporal transcriptome and metabolite analyses provide insights into the biochemical and physiological processes underlying endodormancy release in pistachio (Pistacia vera L.) flower buds
Source: Front Plant Sci. 2023 Sep 22;14:1240442. doi: 10.3389/fpls.2023.1240442 (PMC10556704; doi:10.3389/fpls.2023.1240442)
Supplement: Supplementary file 5 [file Presentation_2.pdf]

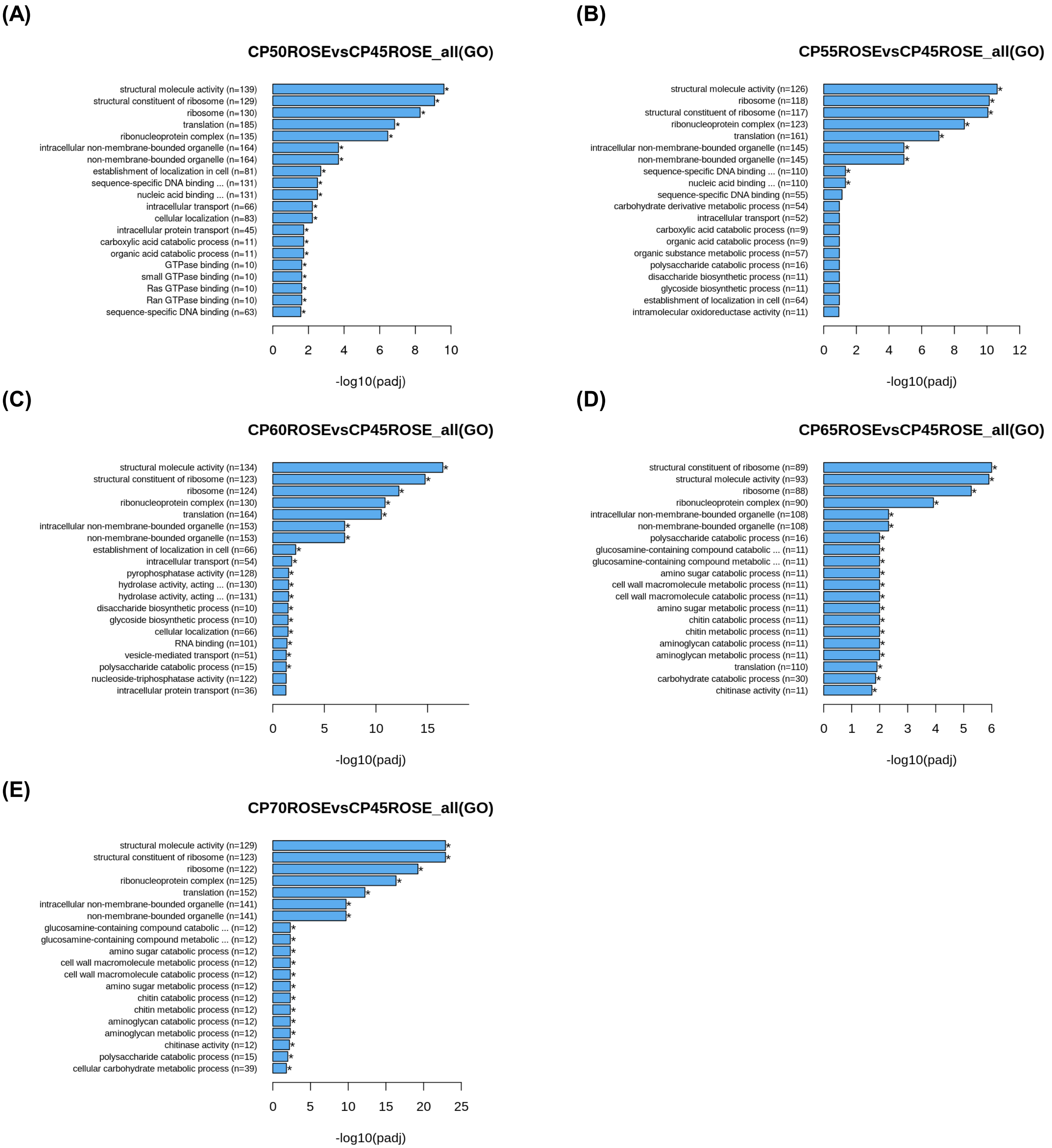

Figure S2. Gene ontology (GO) enrichment histograms for differentially expressed genes in buds collected at the Rose orchard. Top 20 significantly enriched terms in the GO enrichment analysis are shown.
