# Supplementary material for: Temporal transcriptome and metabolite analyses provide insights into the biochemical and physiological processes underlying endodormancy release in pistachio (Pistacia vera L.) flower buds
Source: Front Plant Sci. 2023 Sep 22;14:1240442. doi: 10.3389/fpls.2023.1240442 (PMC10556704; doi:10.3389/fpls.2023.1240442)
Supplement: Supplementary file 8 [file Presentation_5.pdf]

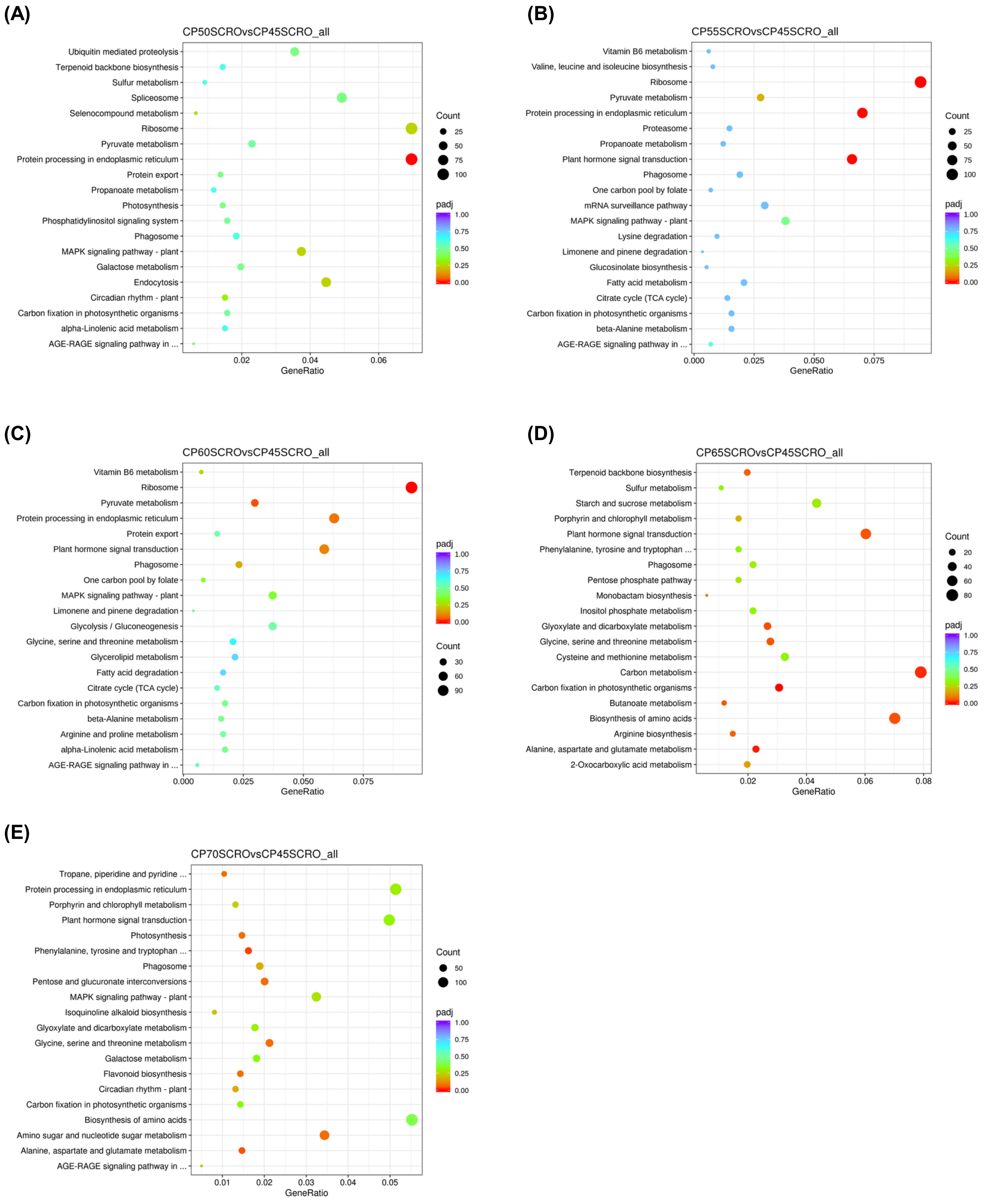

Figure S5. Kyoto Encyclopedia of Genes and Genomes (KEGG) pathway enrichment analysis for differentially expressed genes (DEGs) in buds collected at the Scroggs orchard. The size of the dot indicates the number of DEGs in the specific pathway, whereas the color of the dot represents the adjusted *p* value of the enrichment gene ratio (DEGs in the specific pathway/total DEGs).
