# Supplementary material for: Temporal transcriptome and metabolite analyses provide insights into the biochemical and physiological processes underlying endodormancy release in pistachio (Pistacia vera L.) flower buds
Source: Front Plant Sci. 2023 Sep 22;14:1240442. doi: 10.3389/fpls.2023.1240442 (PMC10556704; doi:10.3389/fpls.2023.1240442)
Supplement: Supplementary file 9 [file Presentation_6.pdf]

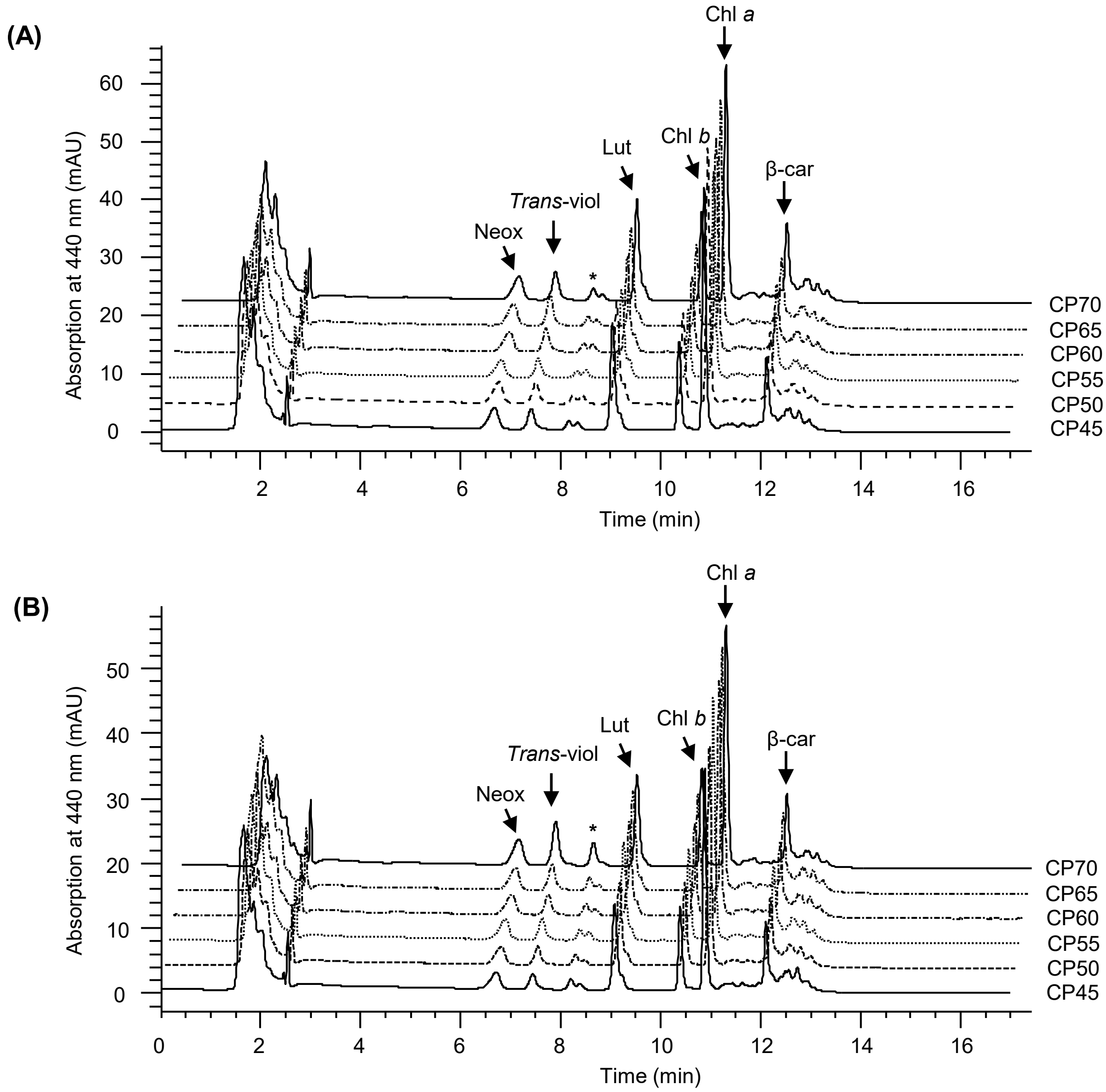

Figure S6. Carotenoid profiles of pistachio buds collected at the Rose (A) and Scroggs (B) orchards. HPLC chromatograms of carotenoids extracted from pistachio buds exposed to different chill portions (CPs). Neox, neoxanthin; *trans*-viol, *trans*-violaxanthin; Lut, lutein; Chl *b*, chlorophyll *b*; Chl *a*, chlorophyll *a*; β-car, β-carotene. * indicates an unidentified carotenoid.
