# Supplementary material for: Temporal transcriptome and metabolite analyses provide insights into the biochemical and physiological processes underlying endodormancy release in pistachio (Pistacia vera L.) flower buds
Source: Front Plant Sci. 2023 Sep 22;14:1240442. doi: 10.3389/fpls.2023.1240442 (PMC10556704; doi:10.3389/fpls.2023.1240442)
Supplement: Supplementary file 10 [file Presentation_7.pdf]

(A)

```
EVM0022601.1      ATGCTGCTGGATAAGCTTTGGGACGACGGCGTTGGAGGCCCTCACCCTGAACGTGGCCTTGGCAAGCTCAGGAAGATCAC
EVM0002551.1      ATGCTGCTGGATAAGCTTTGGGACGACGGCGTTGGAGGCCCTCACCCTGAACGTGGCCTTGGCAAGCTCAGGAAGATCAC
                  ********************************************************************************

EVM0022601.1      CACTCAACCCTTGAACATCAAAGATGGTGAAGGAGAGTCAAGCAGGTACCAGAGATCTCTCTCAATGCCCGCAAGTCCAG
EVM0002551.1      CACTCAACCCTTGAACATCAAAGATGGTGAAGGAGAGTCAAGCAGGTACCAGAGATCTCTCTCAATGCCCGCAAGTCCAG
                  ********************************************************************************

EVM0022601.1      CGACACCGACGACTCCAGTGACACCAACGACACCAGTATCGGCGCGTAAGGACAACGTGTGGAGGAGCGTGTTCCACCCG
EVM0002551.1      CGACACCGACAACTCCAGTGACGCCAACGACACCGGTATCGGCGCGTAAGGACAACGTGTGGAGGAGCGTCTTCCACCCG
                  ********** *********** *********** *********************************** *********

EVM0022601.1      GGTAGCAACCTTGCCACTAGAGGCATCGGGGCTGAGGTGTTCGACAAGCCCGTCCATCCCAACTCCCCCTCTGTTTATGA
EVM0002551.1      GGTAGCAACCTTGCCACTAGAGGCATCGGGGCTGAGGTGTTCGACAAGCCCGTCCATCCCAACTCCCCCTCTGTTTATGA
                  ********************************************************************************

EVM0022601.1      CTGGCTCTACAGTGGCGAGACAAGGAGCAAGAACCACCATCACTAA
EVM0002551.1      CTGGCTCTACAGTGGCGAGACAAGGAGCAAGAACCACCATCACTAA
                  **********************************************
```

(B)

>EVM0002551 (DAM)

MLLDK<u>LWDDGVGGPHPERGLGKLRKITTQPLNIKDGEGESSRYQRSLSMPASPATPTTPVTPTTPVSARKDNVWRSVF</u>
<u>HPGSNLATRGIGAEVFDKPVHPNSPSVYDWLYSGETRSKNH</u>HH

>EVM0022601 (DAM)

MLLDK<u>LWDDGVGGPHPERGLGKLRKITTQPLNIKDGEGESSRYQRSLSMPASPATPTTPVTPTTPVSARKDNVWRSVF</u>
<u>HPGSNLATRGIGAEVFDKPVHPNSPSVYDWLYSGETRSKNH</u>HHH

>EVM0027471 (DAM)

MSLLDQ<u>LWDDTLAGPRPESGLGKLRKHSTFSFRPNSGKESNGGNVRSDVDESPLEAAKVTRSIMIVKPPGYQNGSPPV</u>
<u>SPAGSTP</u>PVSPFSGKPKLLVQMTNDLLFQVPKMPSPIIMEFVITGINQRKARIYLSFSAQ

>EVM0028749 (DAM)

MGFLHK<u>LWDETLAGPAPETGLGKLRKYDSFSGTRSPHSPAVNGADDVVVTRSITILRSNSNFRSLSLDPGSAPESPAG</u>
<u>PDTPRTPKTPGTP</u>GGDFKKFTRRKLSTVALERDADEPRIL

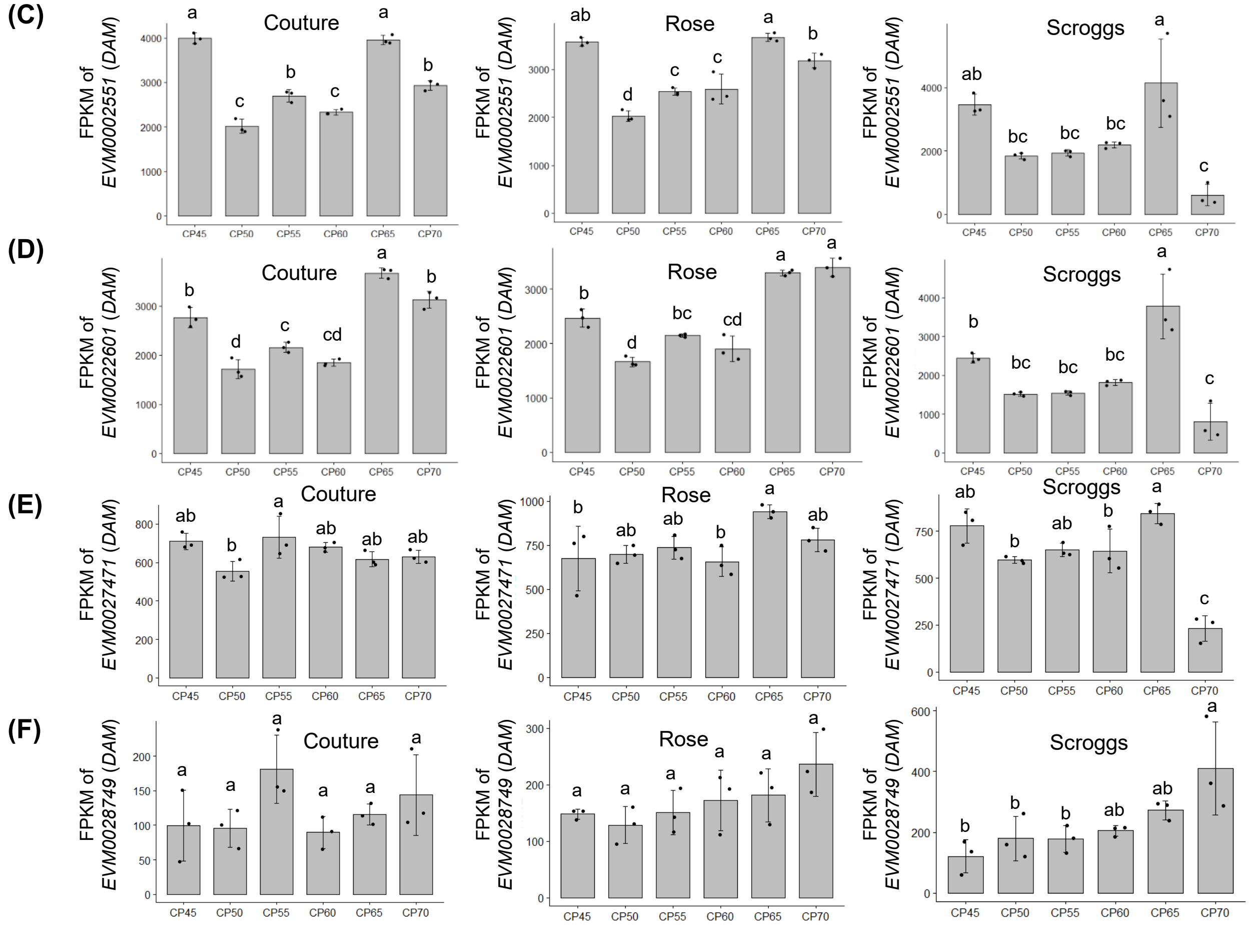

Figure S7. Expression and sequence analyses of the pistachio homologs of *Dormancy Associated MADS-box* (*DAM*) genes *EVM0002551*, *EVM0022601*, *EVM0027471*, and *EVM0028749*. (A) Nucleotide sequence alignment of *EVM0002551* and *EVM0022601*. (B) The deduced amino acid sequences of EVM0002551, EVM0022601, EVM0027471, and EVM0028749. The MADS domain is underlined. The transcript levels of *EVM0002551* (C), *EVM0022601* (D), *EVM0027471* (E), and *EVM0028749* (F) were determined by RNA-Seq analysis. Bud tissues were collected at three orchards: Couture, Rose, and Scroggs. Values are means with SD from three biological replicates. Different letters indicate statistically significant differences ($p < 0.05$) determined by Tukey's Honest Significant Difference test. FPKM, Fragments Per Kilobase of transcript per Million mapped reads; CP, chill portion.
